# Supplementary material for: New Napyradiomycin Analogues from Streptomyces sp. Strain CA-271078
Source: Mar Drugs. 2019 Dec 26;18(1):22. doi: 10.3390/md18010022 (PMC7024253; doi:10.3390/md18010022)
Supplement: Supplementary file 1 [file marinedrugs-18-00022-s001.pdf]

Supplementary Materials

New Napyradiomycin Analogues from *Streptomyces* sp. Strain CA-271078

Daniel Carretero-Molina [1,2], Francisco Javier Ortiz-López [1,*], Jesús Martín [1], Daniel Oves-Costales [1], Caridad Díaz [1], Mercedes de la Cruz [1], Bastien Cautain [1], Francisca Vicente [1], Olga Genilloud [1] and Fernando Reyes [1,*]

[1] Fundación MEDINA, Centro de Excelencia en Investigación de Medicamentos Innovadores en Andalucía, Avda. del Conocimiento 34, 18016, Armilla (Granada), Spain; daniel.carretero@medinaandalucia.es (D.C.-M.); jesus.martin@medinaandalucia.es (J.M.); daniel.oves@medinaandalucia.es (D.O.-C.); caridad.diaz@medinaandalucia.es (C.D.); mercedes.delacruz@medinaandalucia.es (M.d.l.C.); bastien.cautain@medinaandalucia.es (B.C.); francisca.vicente@medinaandalucia.es (F.V.); olga.genilloud@medinaandalucia.es (O.G.)

[2] Doctoral Programme in Pharmacy (B15.56.1), Doctoral School in Health Sciences, University of Granada, Spain.

* Correspondence: javier.ortiz@medinaandalucia.es (F.J.O.-L.); fernando.reyes@medinaandalucia.es (F.R.); Tel.: +34-958-993-965 (F.J.O.-L. and F.R.)

Received: 10 December 2019; Accepted: 25 December 2019; Published: date

List of Supplementary Materials:

Figure S1. Electrospray-time of flight (ESI-TOF) (**A**) and UV (**B**) spectra for compound **1**.
Figure S2. ^{1}H NMR spectrum (DMSO-d_6, 500 MHz) of compound **1**.
Figure S3. ^{13}C NMR spectrum (DMSO-d_6, 125 MHz) of compound **1**.
Figure S4. COSY spectrum of compound **1**.
Figure S5. HSQC spectrum of compound **1**.
Figure S6. HMBC spectrum of compound **1**.
Figure S7. ROESY spectrum of compound **1**.
Figure S8. Energy minimized molecular model of compound **1**.
Figure S9. Electrospray-time of flight (ESI-TOF) (**A**) and UV (**B**) spectra for compound **2**.
Figure S10. ^{1}H NMR spectrum (DMSO-d_6, 500 MHz) of compound **2**.
Figure S11. ^{13}C NMR spectrum (DMSO-d_6, 125 MHz) of compound **2**.
Figure S12. COSY spectrum of compound **2**.
Figure S13. HSQC spectrum of compound **2**.
Figure S14. HMBC spectrum of compound **2**.
Figure S15. NOESY spectrum of compound **2**.
Figure S16. ROESY spectrum of compound **2**.
Figure S17. Energy minimized molecular model of compound **2**.
Figure S18. Electrospray-time of flight (ESI-TOF) (**A**) and UV (**B**) spectra for compound **3**.
Figure S19. ^{1}H NMR spectrum (DMSO-d_6, 500 MHz) of compound **3**.
Figure S20. ^{13}C NMR spectrum (DMSO-d_6, 125 MHz) of compound **3**.
Figure S21. COSY spectrum of compound **3**.

Figure S22. HSQC spectrum of compound **3**.
Figure S23. HMBC spectrum of compound **3**.
Figure S24. NOESY spectrum of compound **3**.
Figure S25. ROESY spectrum of compound **3**.
Figure S26. Energy minimized molecular model of compound **3**.
Figure S27. Electrospray-time of flight (ESI-TOF) (**A**) and UV (**B**) spectra for compound **4**.
Figure S28. ^{1}H NMR spectrum (DMSO-d_6, 500 MHz) of compound **4**.
Figure S29. ^{13}C NMR spectrum (DMSO-d_6, 125 MHz) of compound **4**.
Figure S30. COSY spectrum of compound **4**.
Figure S31. HSQC spectrum of compound **4**.
Figure S32. HMBC spectrum of compound **4**.
Figure S33. NOESY spectrum of compound **4**.
Figure S34. Energy minimized molecular model of compound **4**.
Figure S35. Electrospray-time of flight (ESI-TOF) (**A**) and UV (**B**) spectra for compound **5**.
Figure S36. ^{1}H NMR spectrum (DMSO-d_6, 500 MHz) of compound **5**.
Figure S37. ^{13}C NMR spectrum (DMSO-d_6, 125 MHz) of compound **5**.
Figure S38. COSY spectrum of compound **5**.
Figure S39. (**A**) HSQC spectrum of compound **5**. (**B**)Key overlay HSQC correlations for compound **5** and **10** supporting the etherification of the 18-hydroxy group in **5**.
Figure S40. (**A)** HMBC of compound **5**. (**B**) Key HMBC correlation supporting the the etherification of the 18-hydroxy group in **5.**.
Figure S41. NOESY spectrum of compound **5**.
Figure S42. Overlay of different conformers for compound **5.**
Figure S43. Napyradiomycin metabolites isolated from CA-271078 (**1**-**15**).

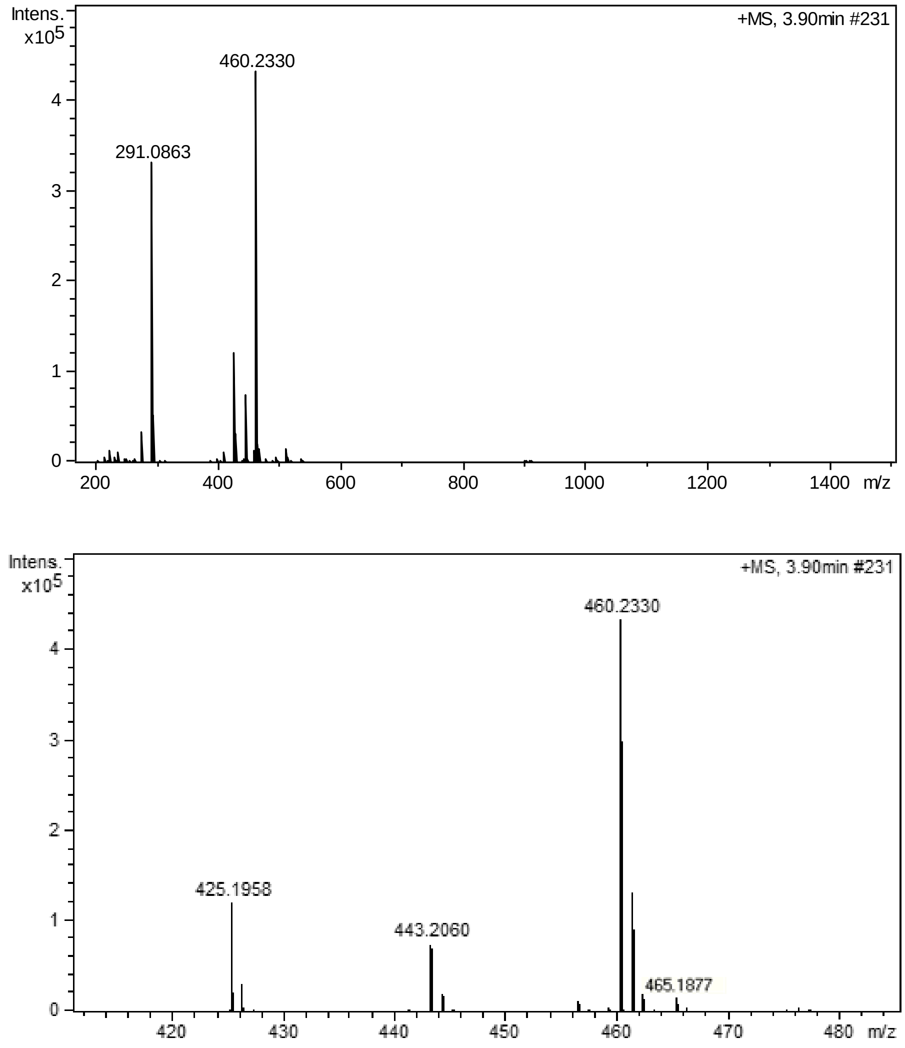

(**A**)

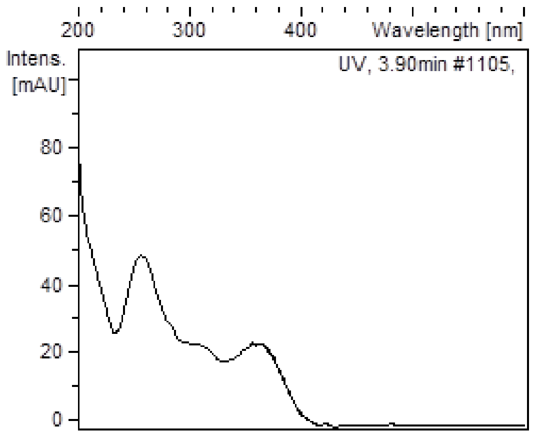

(**B**)

Figure S1. Electrospray-time of flight (ESI-TOF) (**A**) and UV (**B**) spectra of compound **1**.

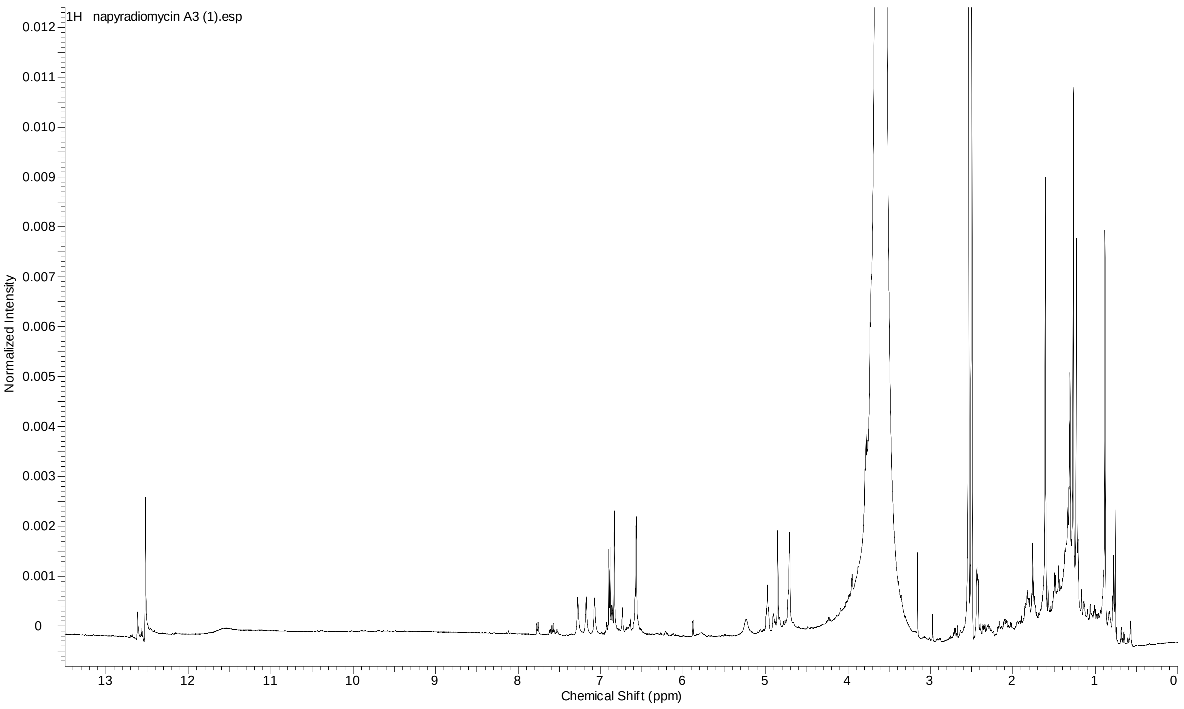

Figure S2. ^{1}H NMR (DMSO_d$_6$, 500 MHz) of compound **1**.

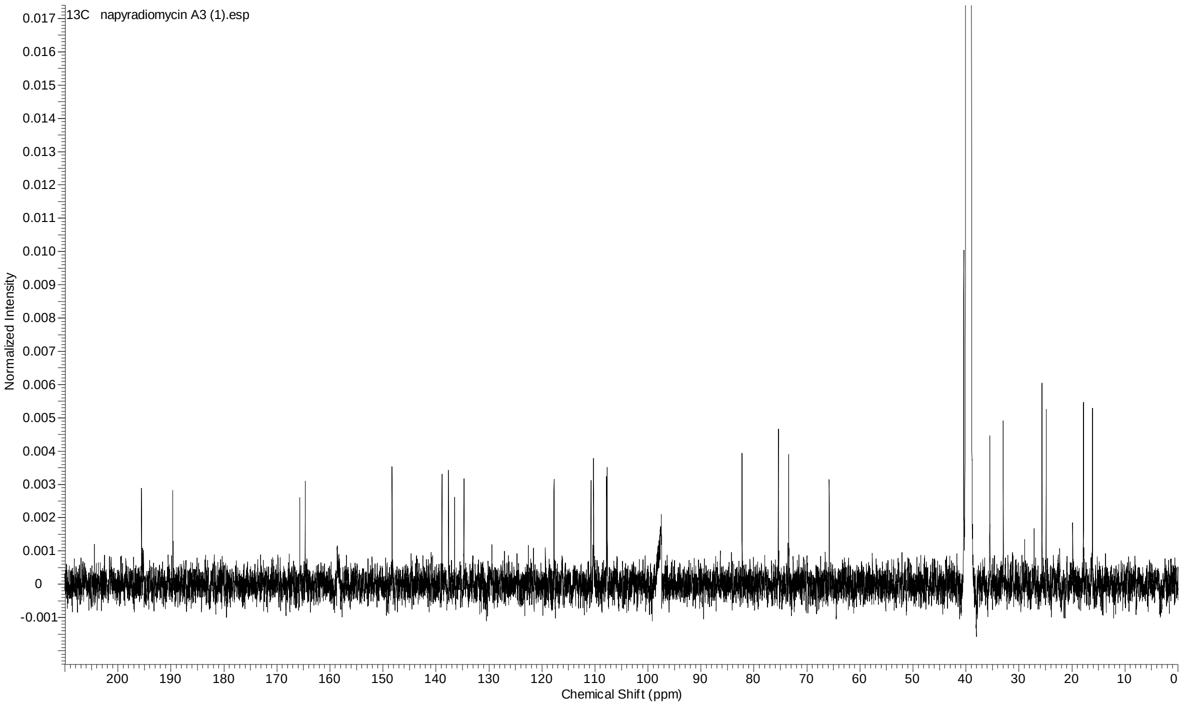

Figure S3. ^{13}C NMR (DMSO_d$_6$, 500 MHz) of compound **1**.

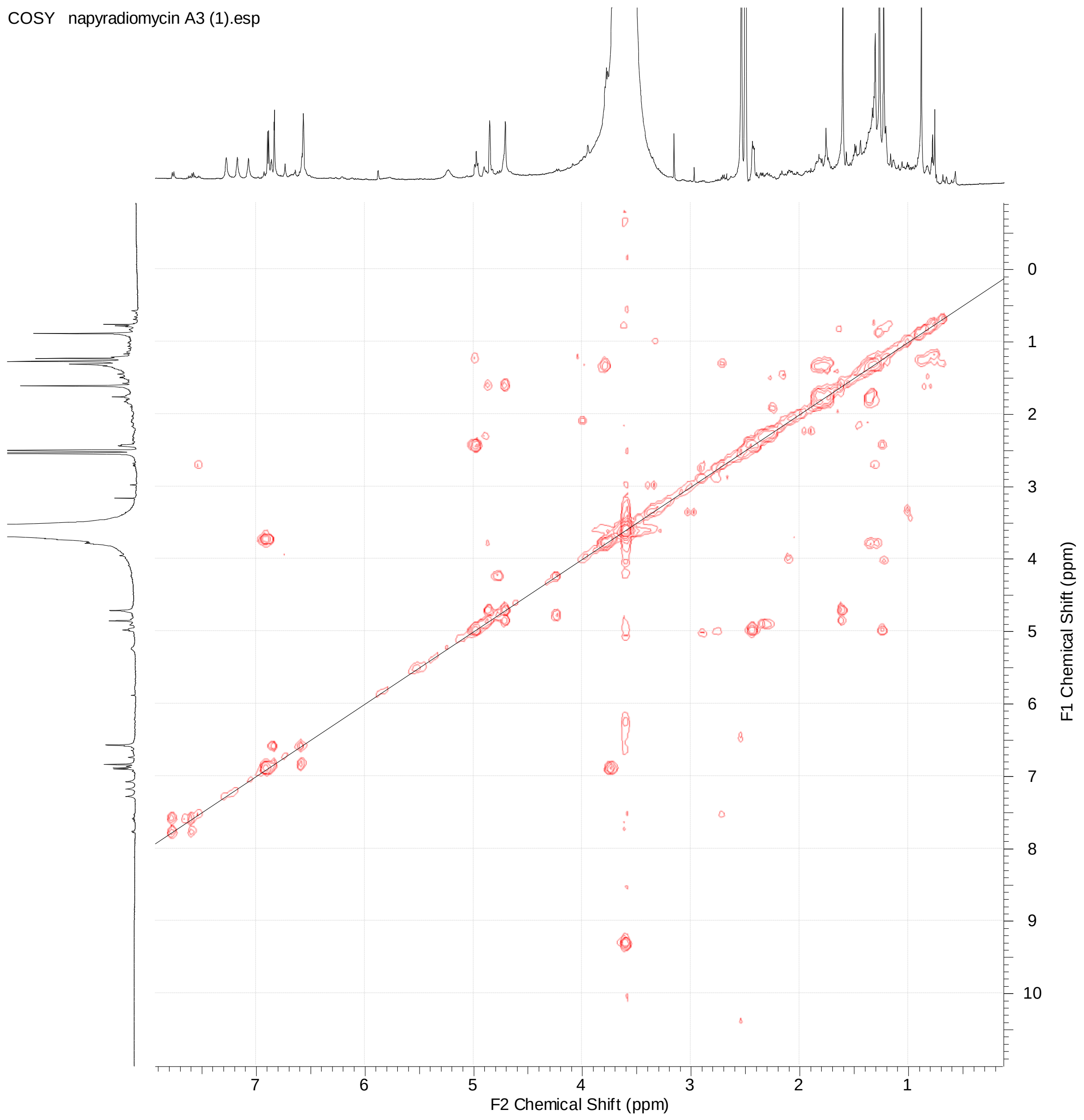

Figure S4. COSY of compound **1**.

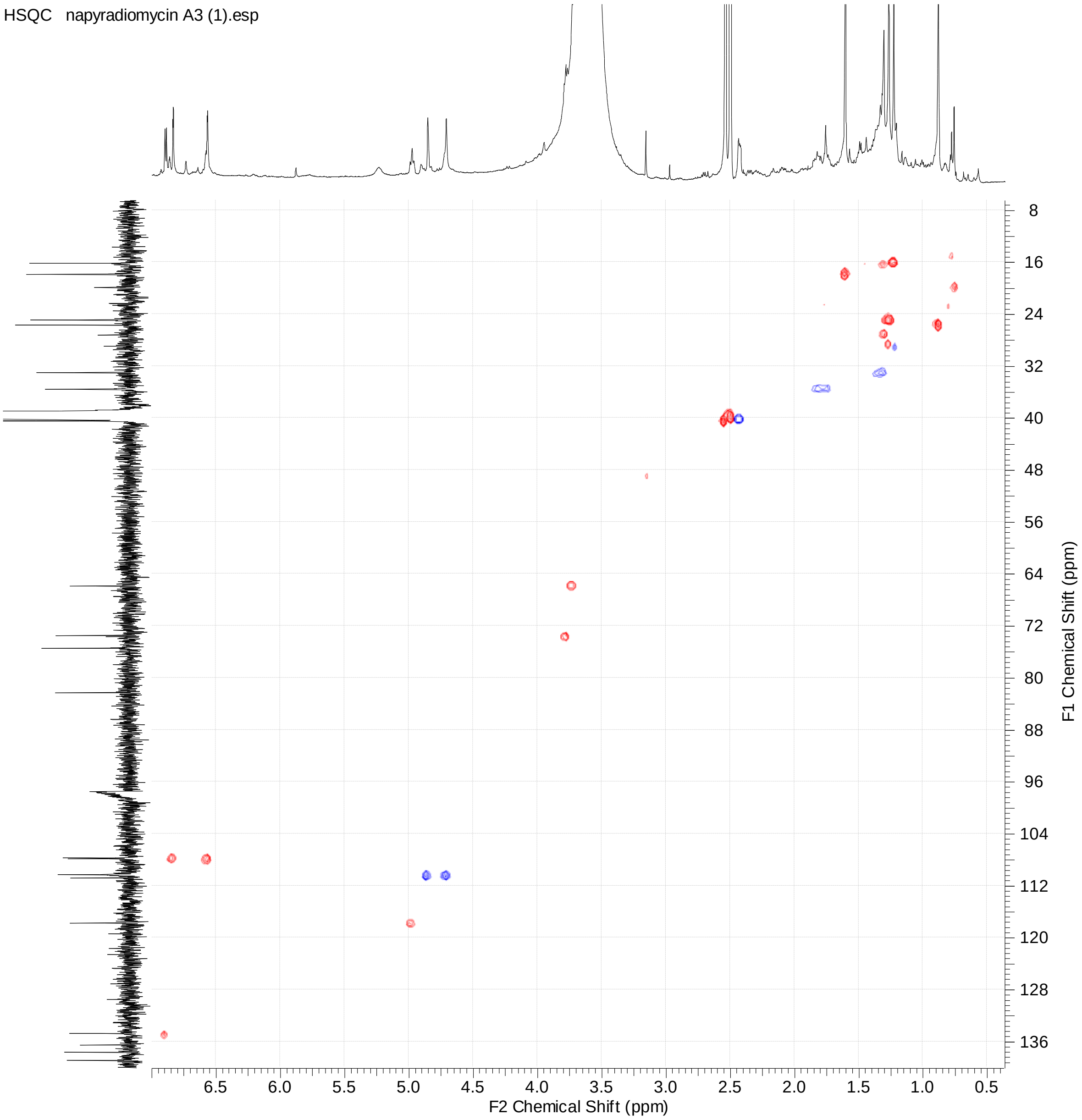

Figure S5. HSQC spectrum of compound **1**.

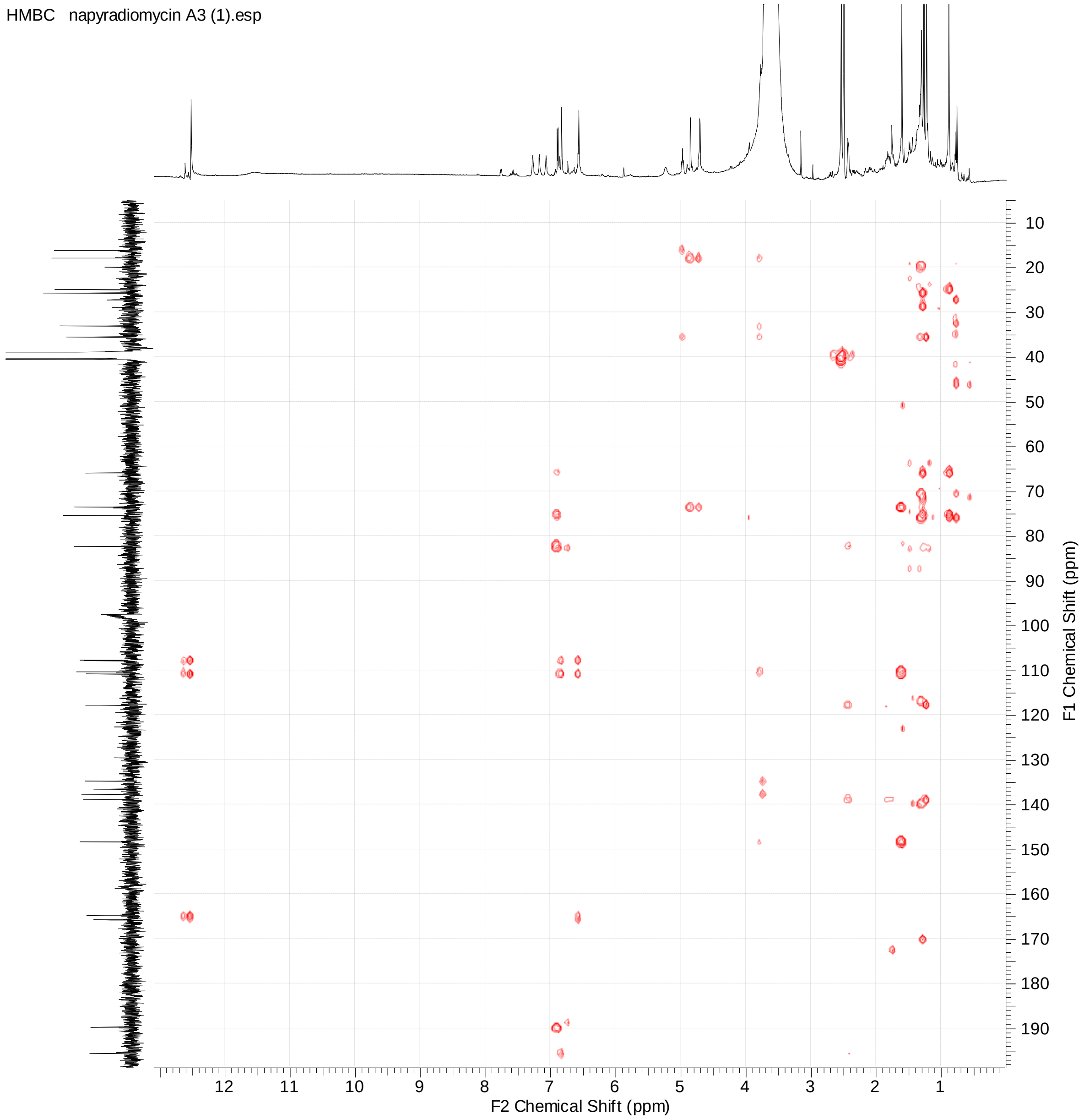

Figure S6. HMBC of compound **1**.

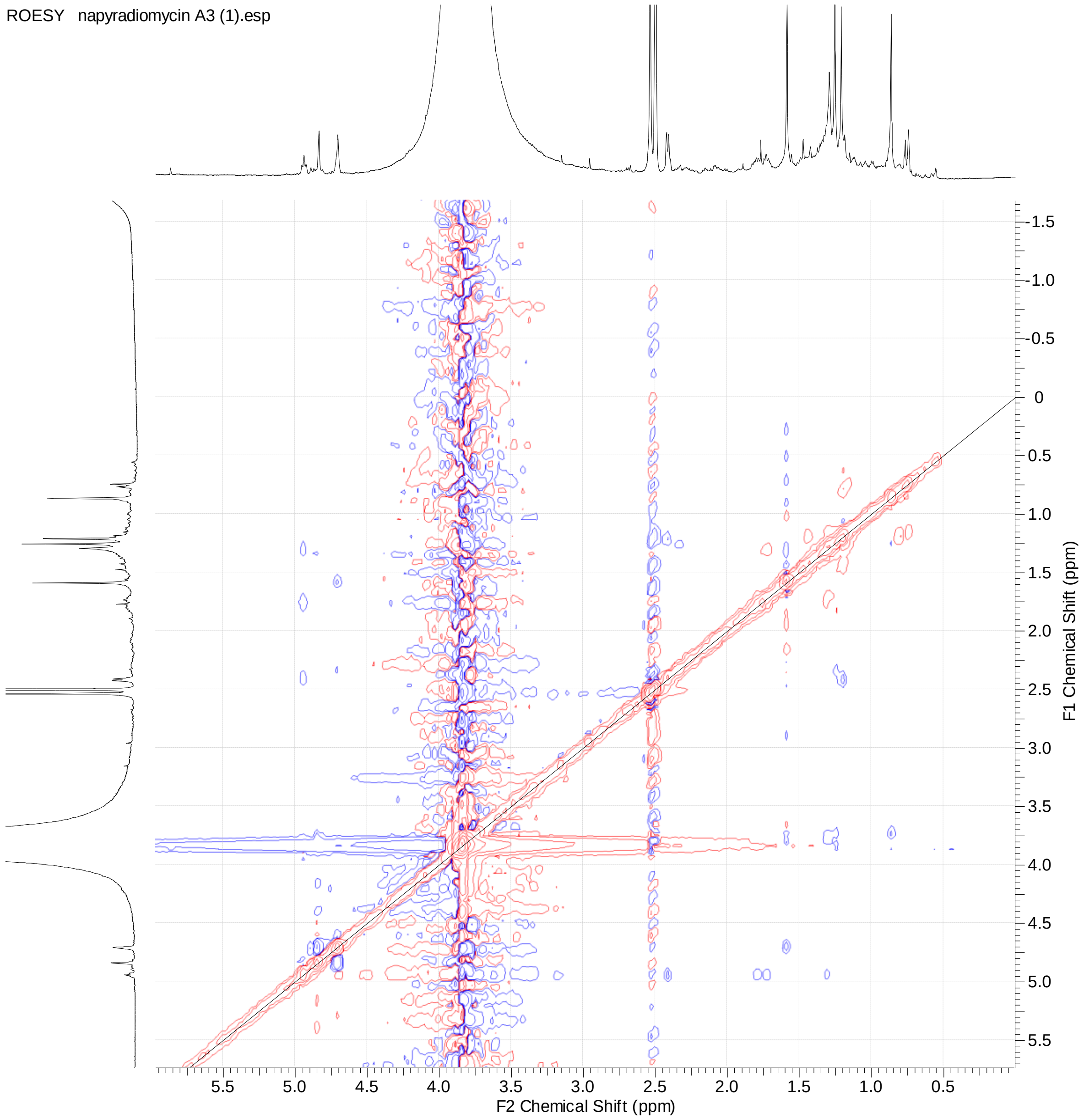

Figure S7. ROESY of compound **1**

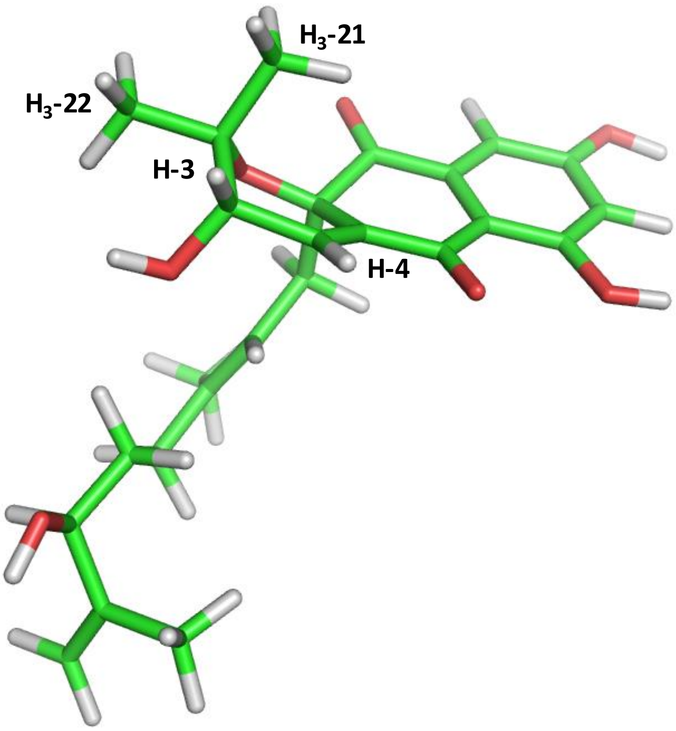

Figure S8. Energy minimized molecular model of compound **1**.

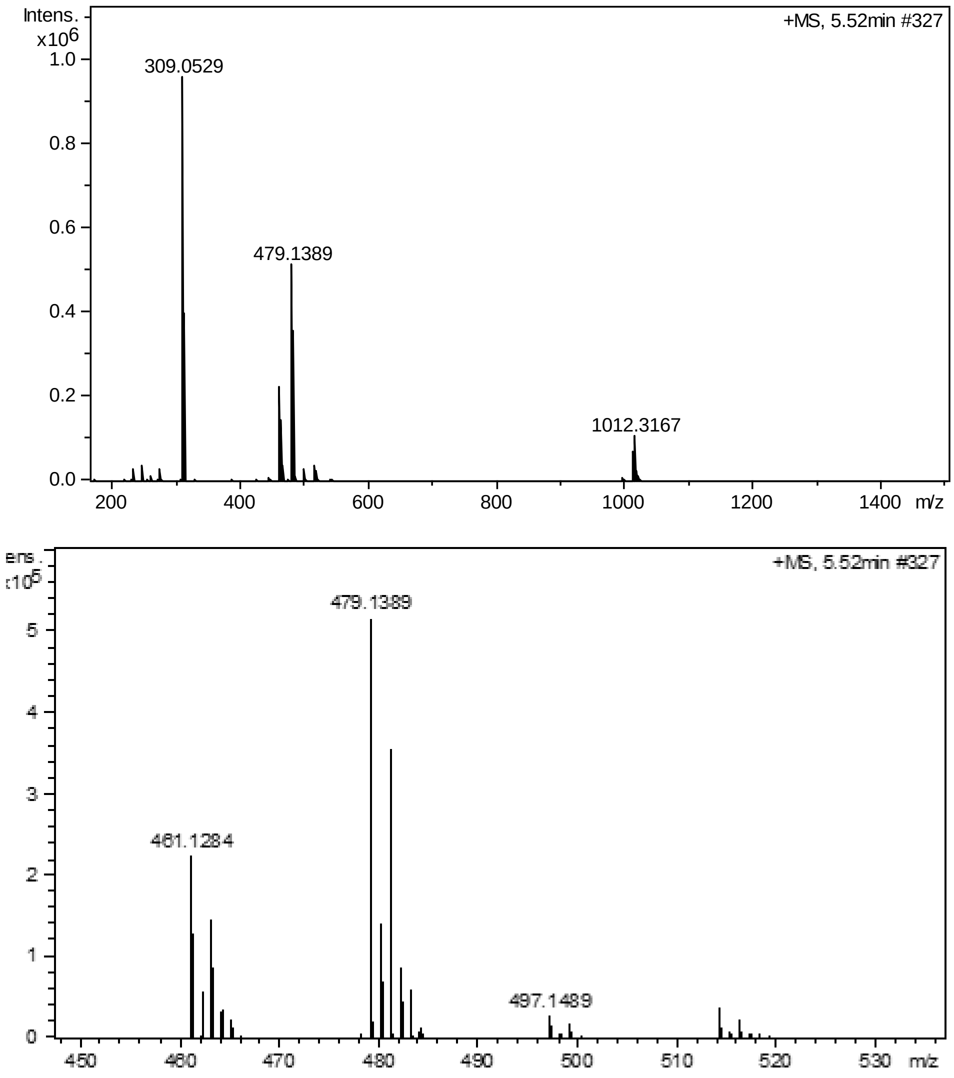

(**A**)

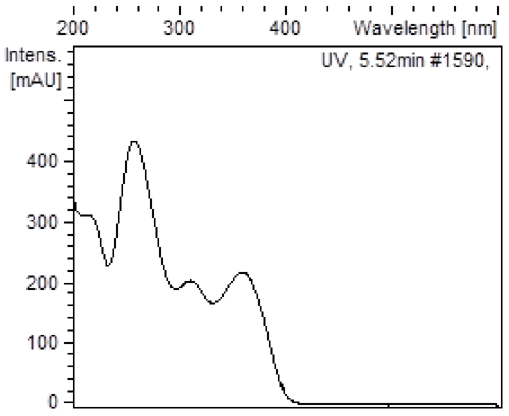

(**B**)

Figure S9. Electrospray-time of flight (ESI-TOF) (**A**) and UV (**B**) spectra of compound **2**.

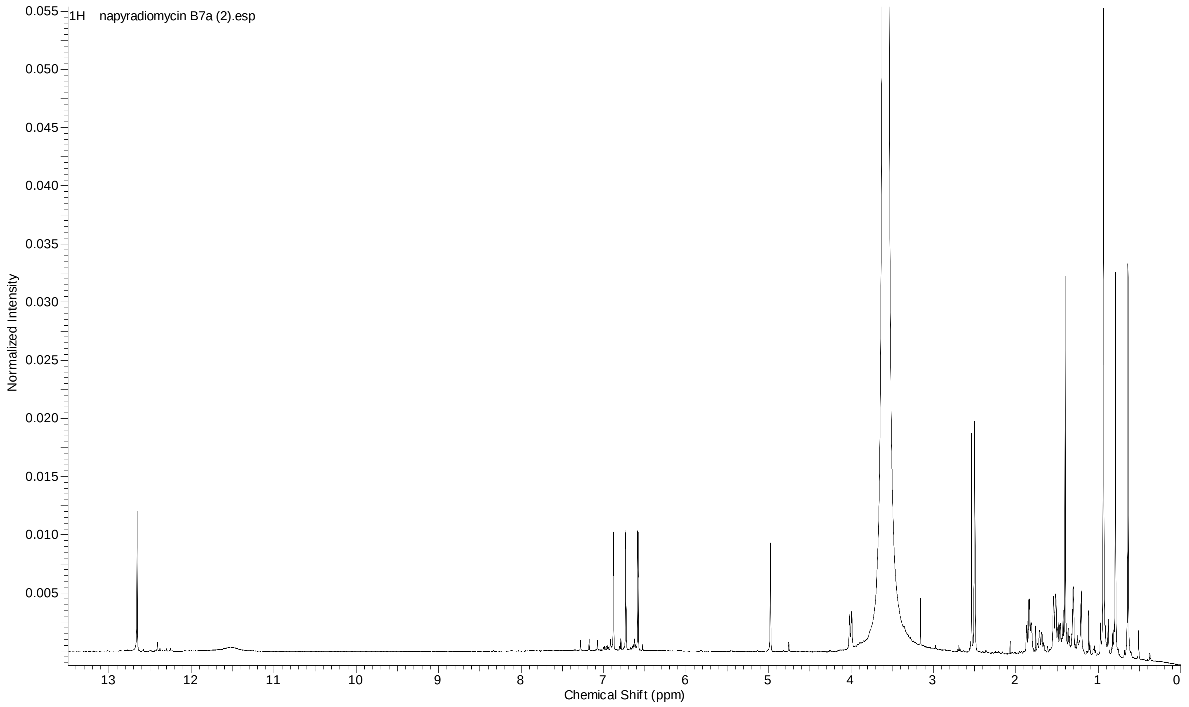

Figure S10. ^{1}H NMR (DMSO_d$_6$, 500 MHz) of compound **2**.

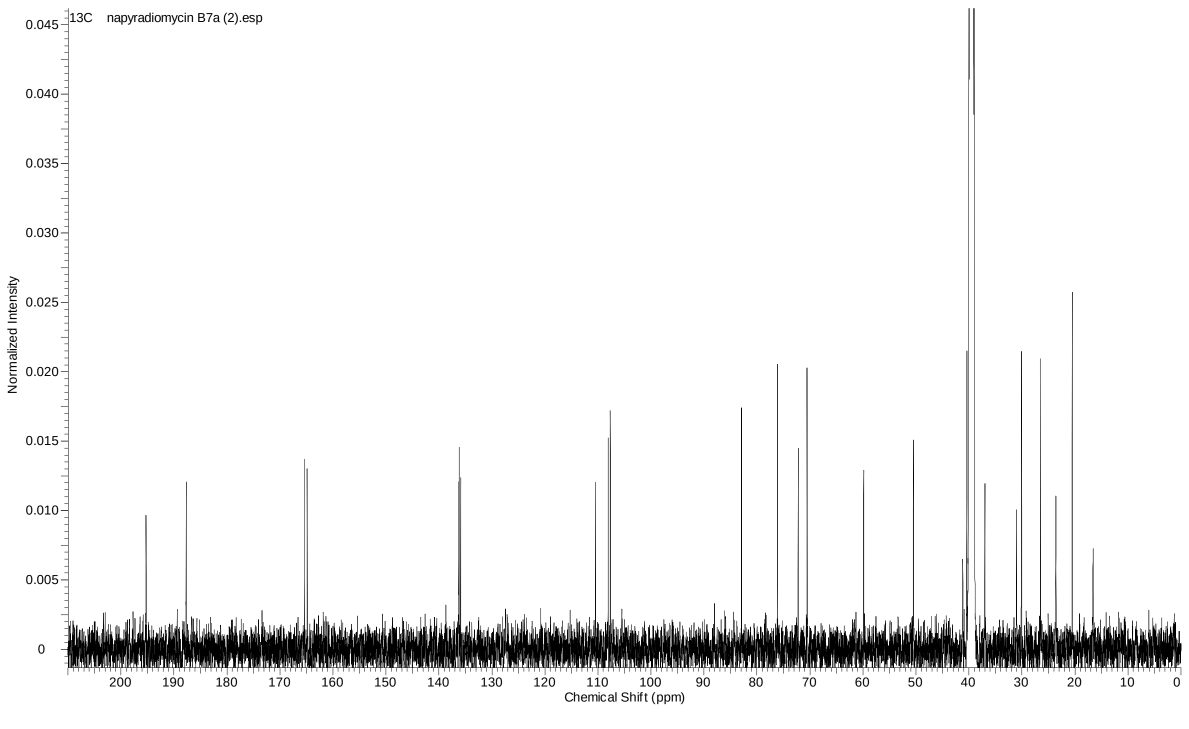

Figure S11. ^{13}C NMR (DMSO_d$_6$, 500 MHz) of compound **2**.

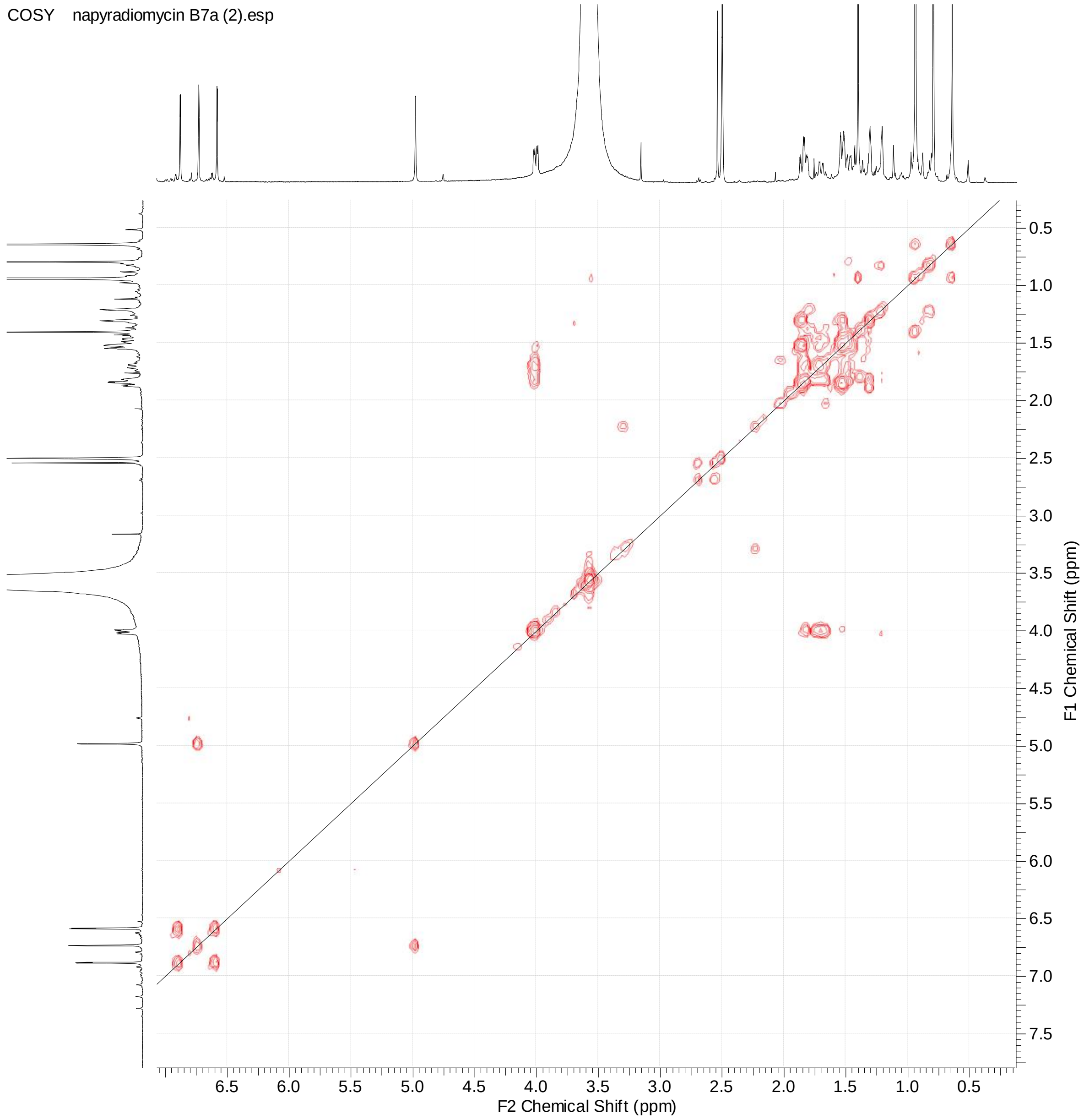

Figure S12. COSY of compound **2**.

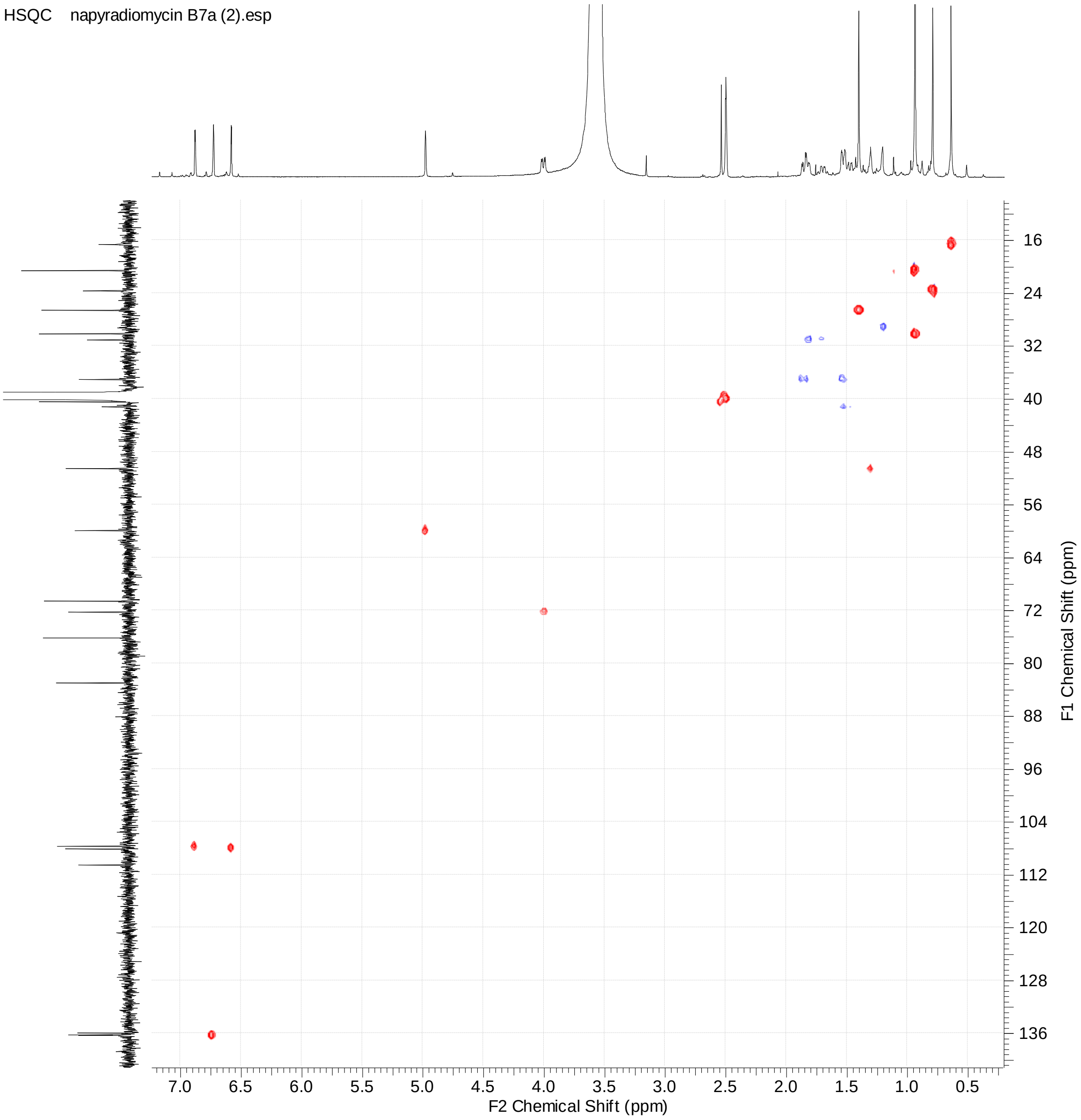

Figure S13. HSQC spectrum of compound **2**.

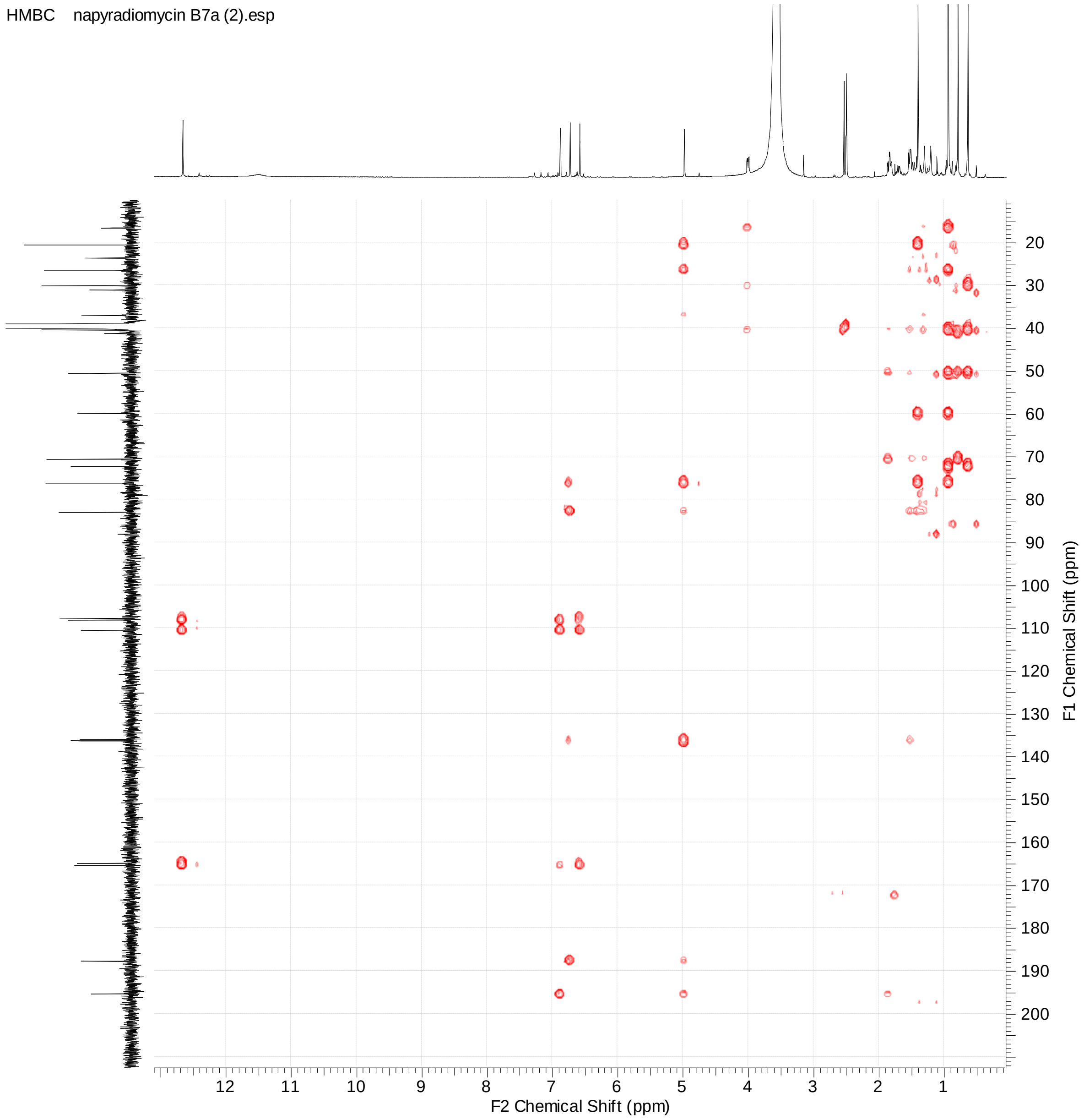

Figure S14. HMBC of compound **2.**

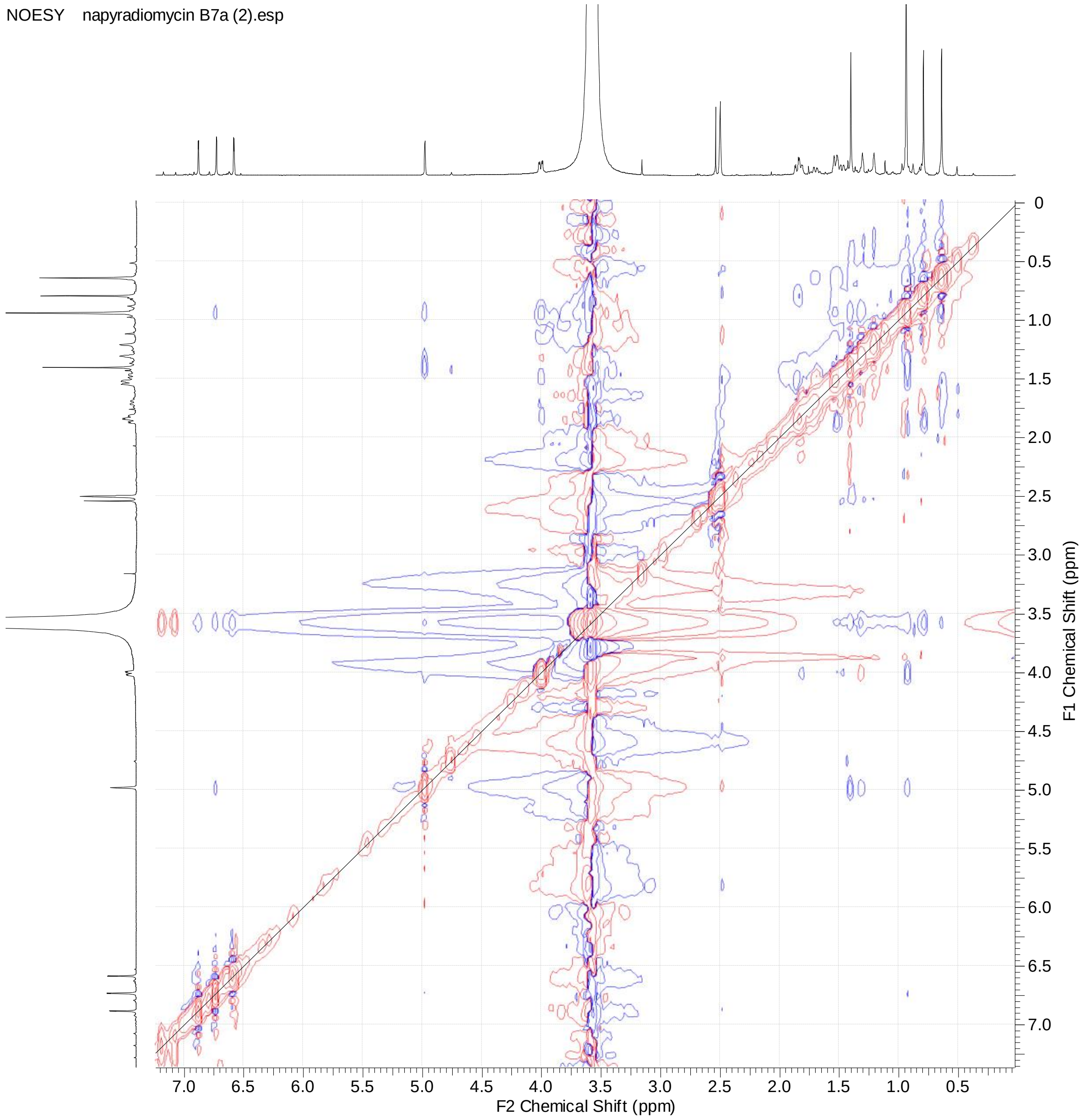

Figure S15. NOESY of compound **2**.

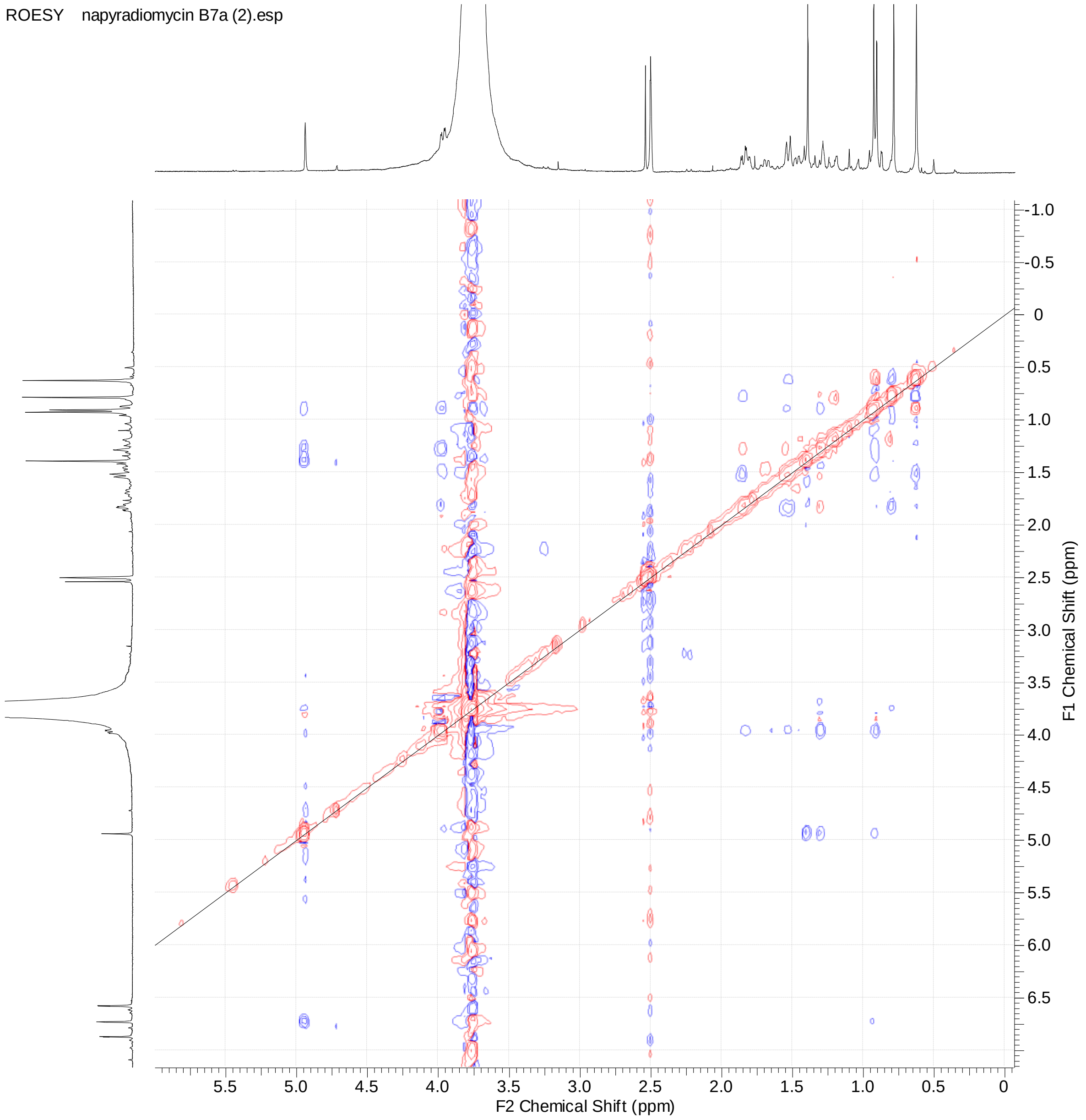

Figure S16. ROESY of compound **2**.

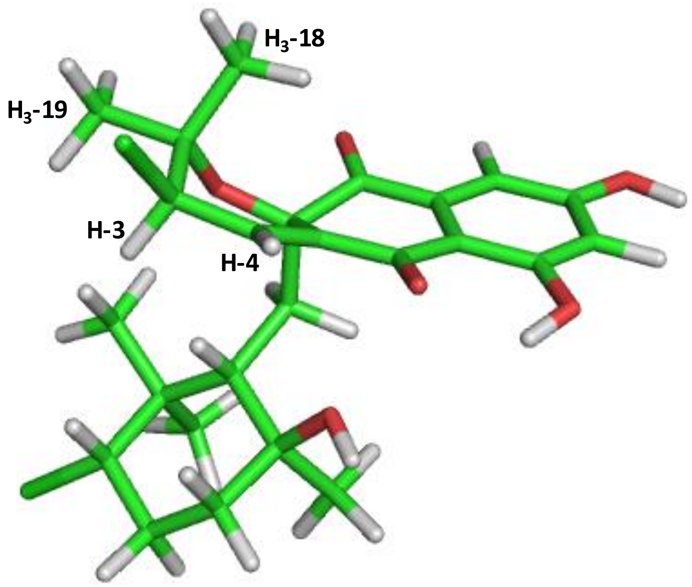

Figure S17. Energy minimized molecular model of compound **2**.

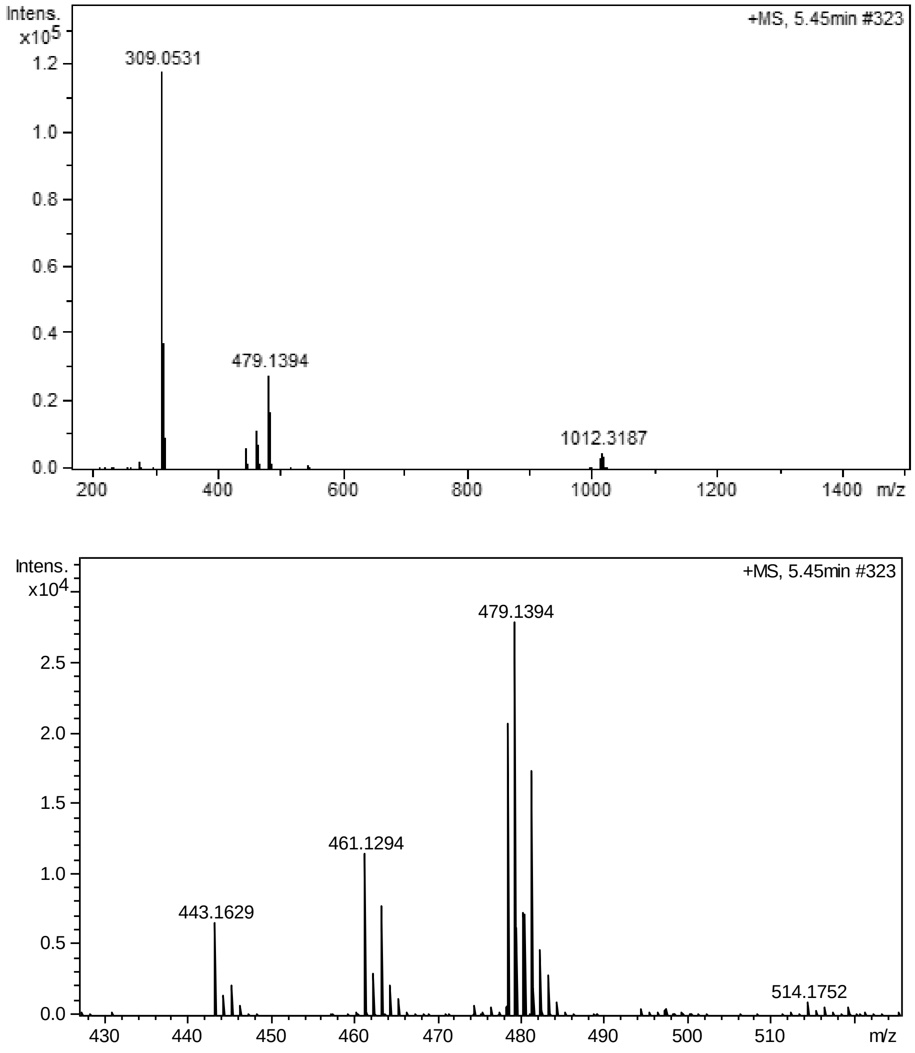

(**A**)

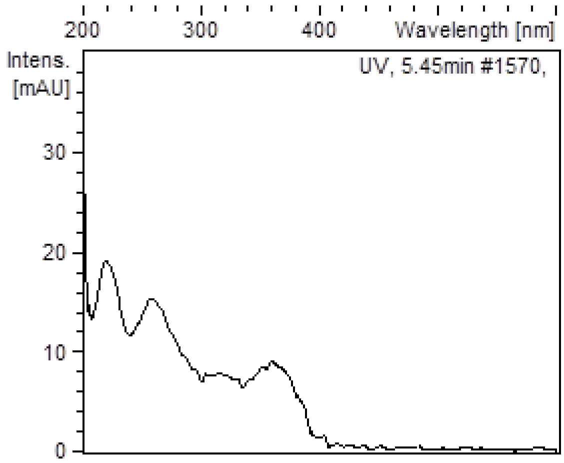

(**B**)

Figure S18. Electrospray-time of flight (ESI-TOF) (**A**) and UV (**B**) spectra of compound **3**.

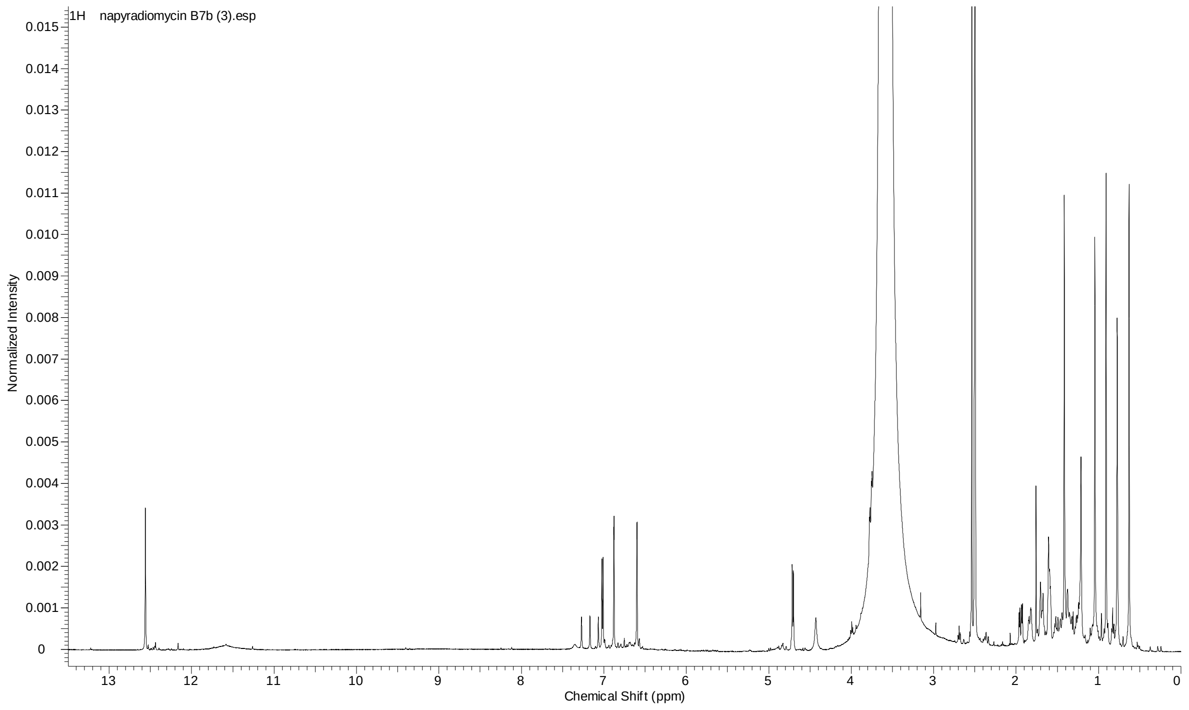

Figure S19. ^{1}H NMR (DMSO_d$_6$, 500 MHz) of compound **3**.

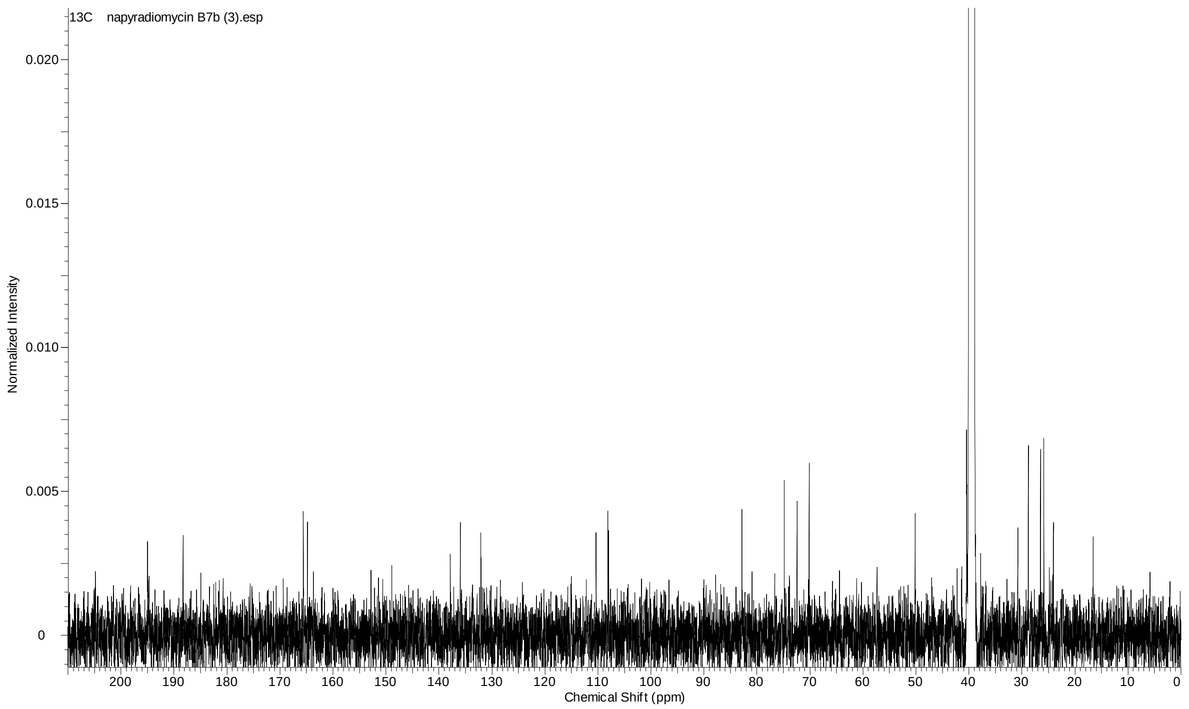

Figure S20. ^{13}C NMR (DMSO_d$_6$, 500 MHz) of compound **3**.

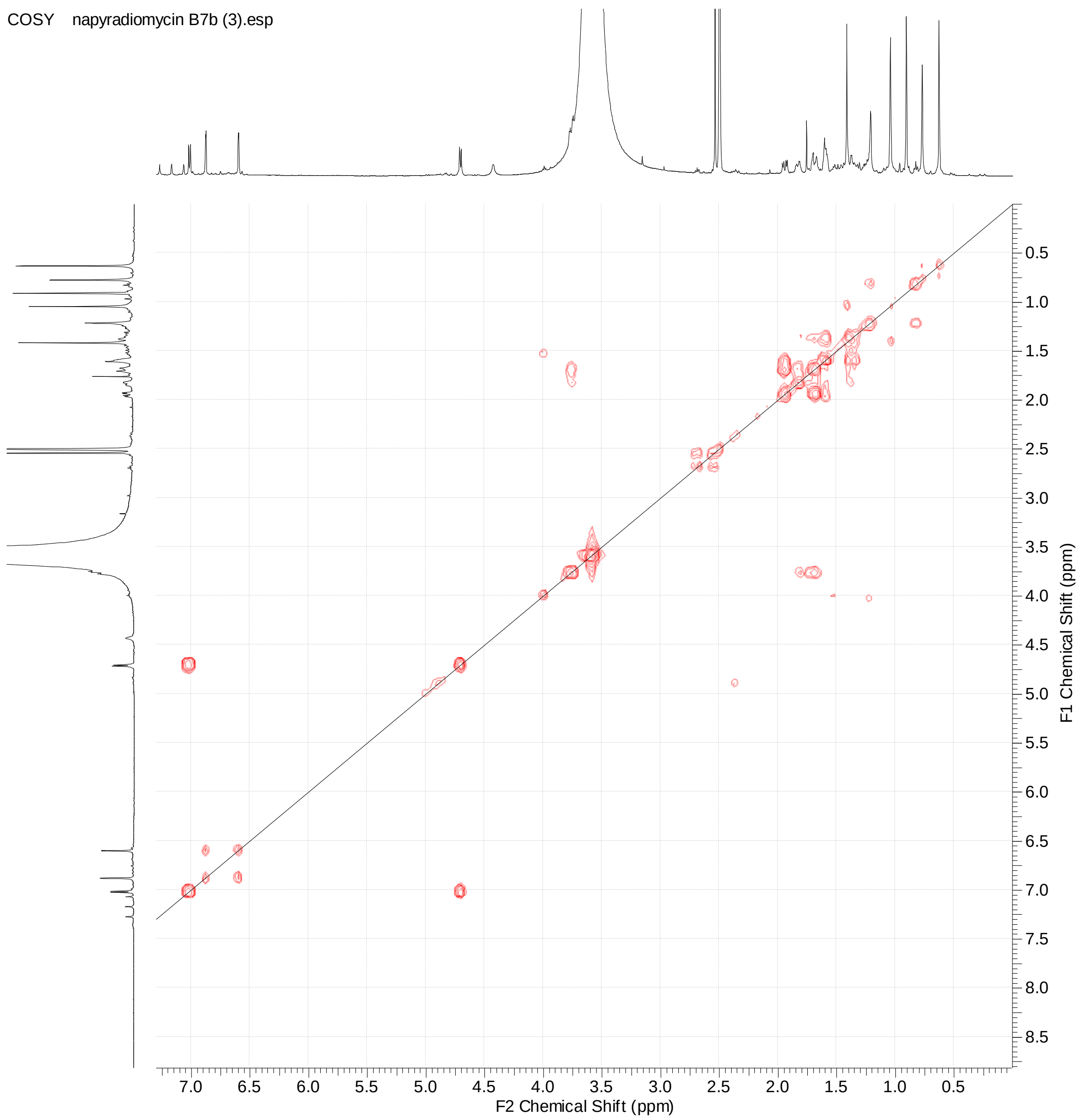

Figure S21. COSY of compound **3**.

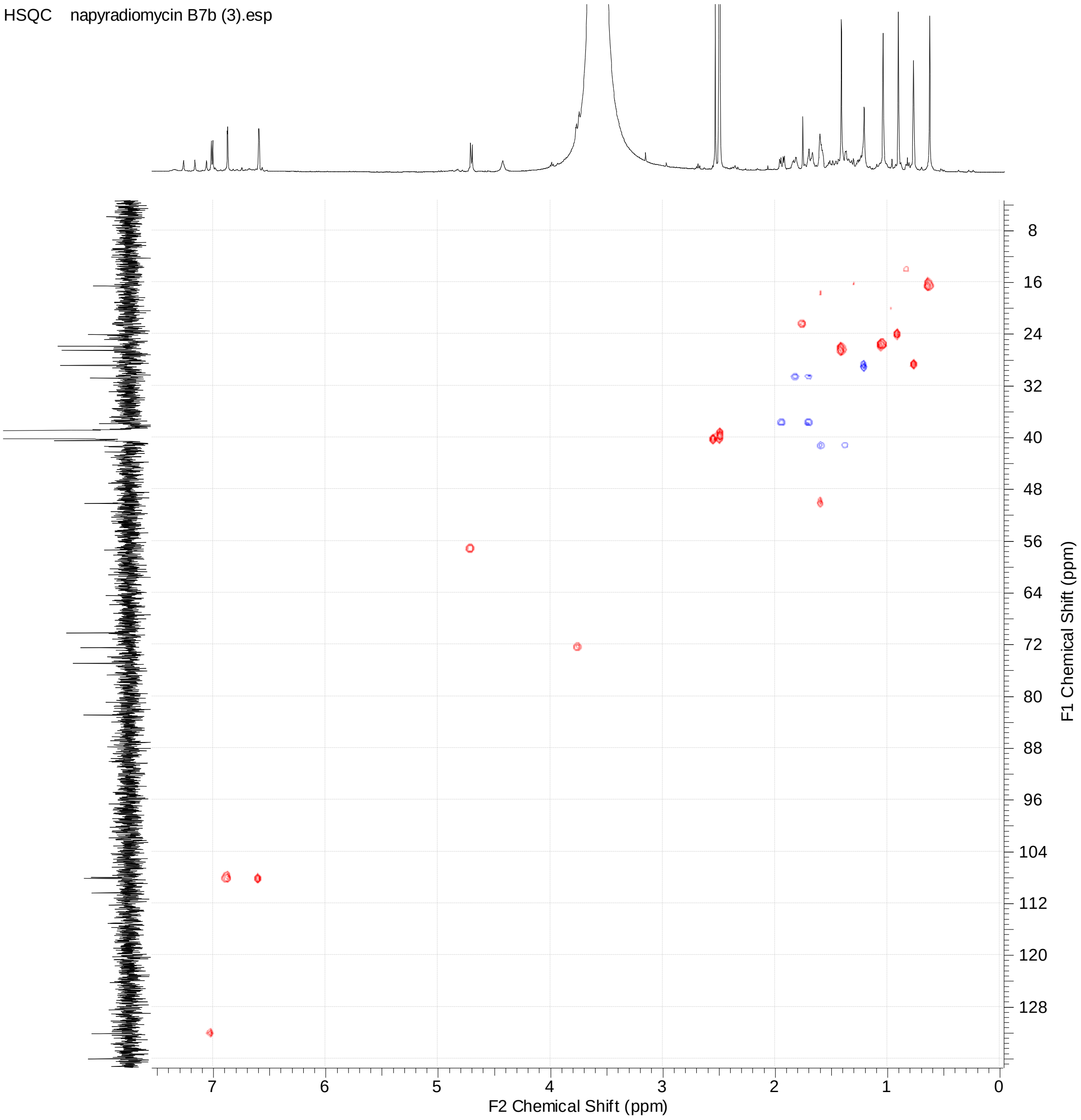

Figure S22. HSQC spectrum of compound **3**.

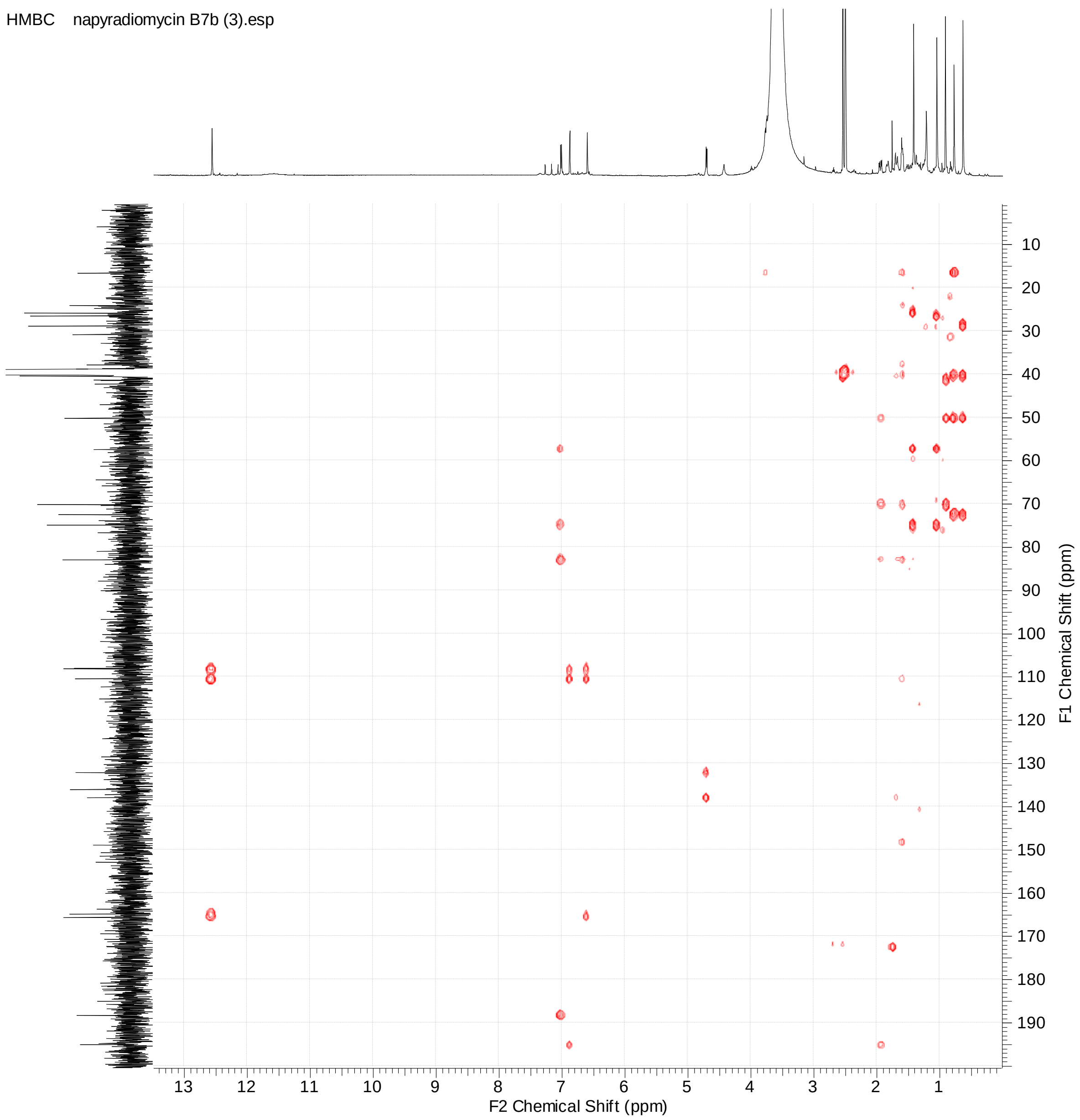

Figure S23. HMBC of compound **3**.

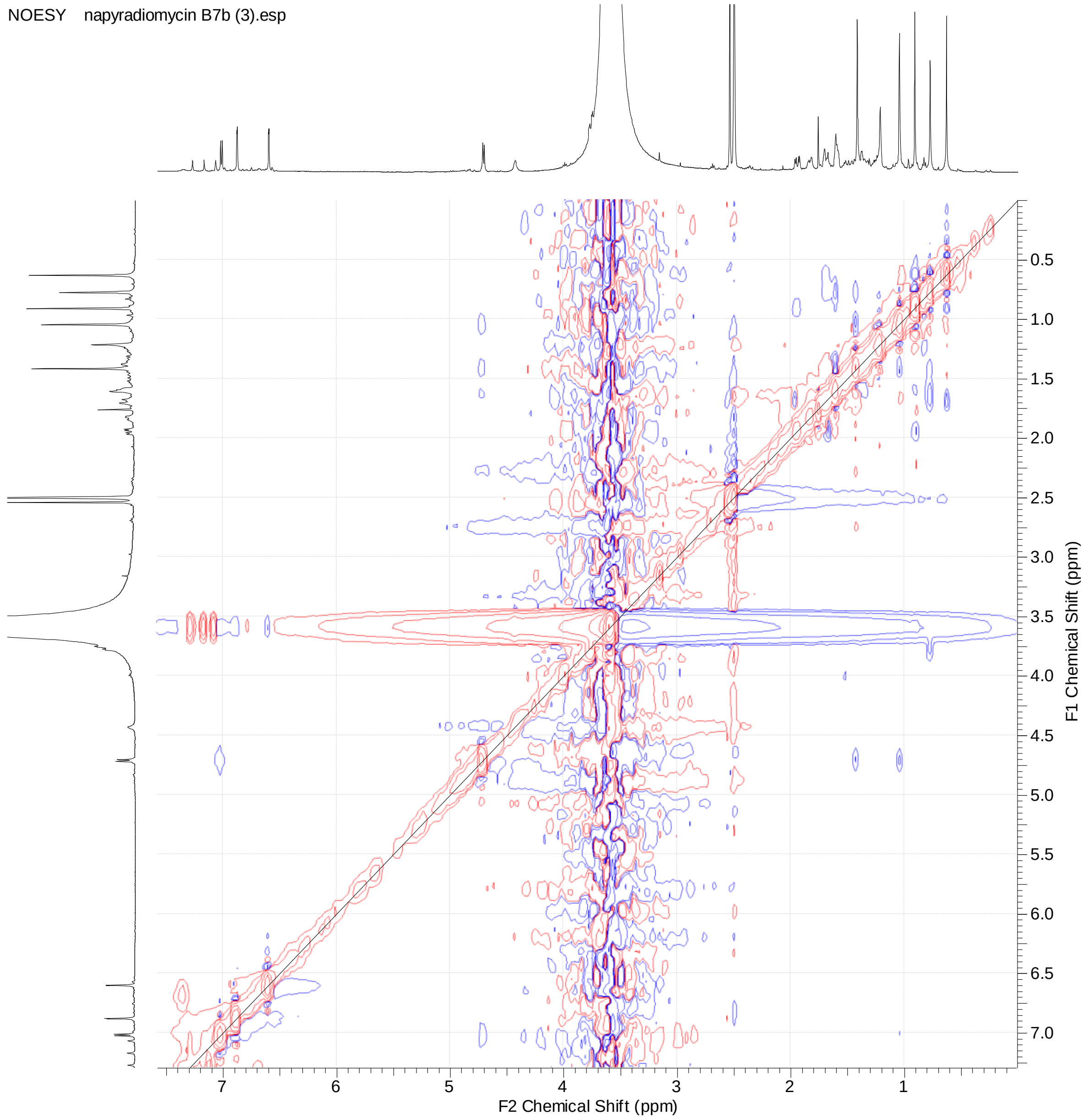

Figure S24. NOESY of compound **3**.

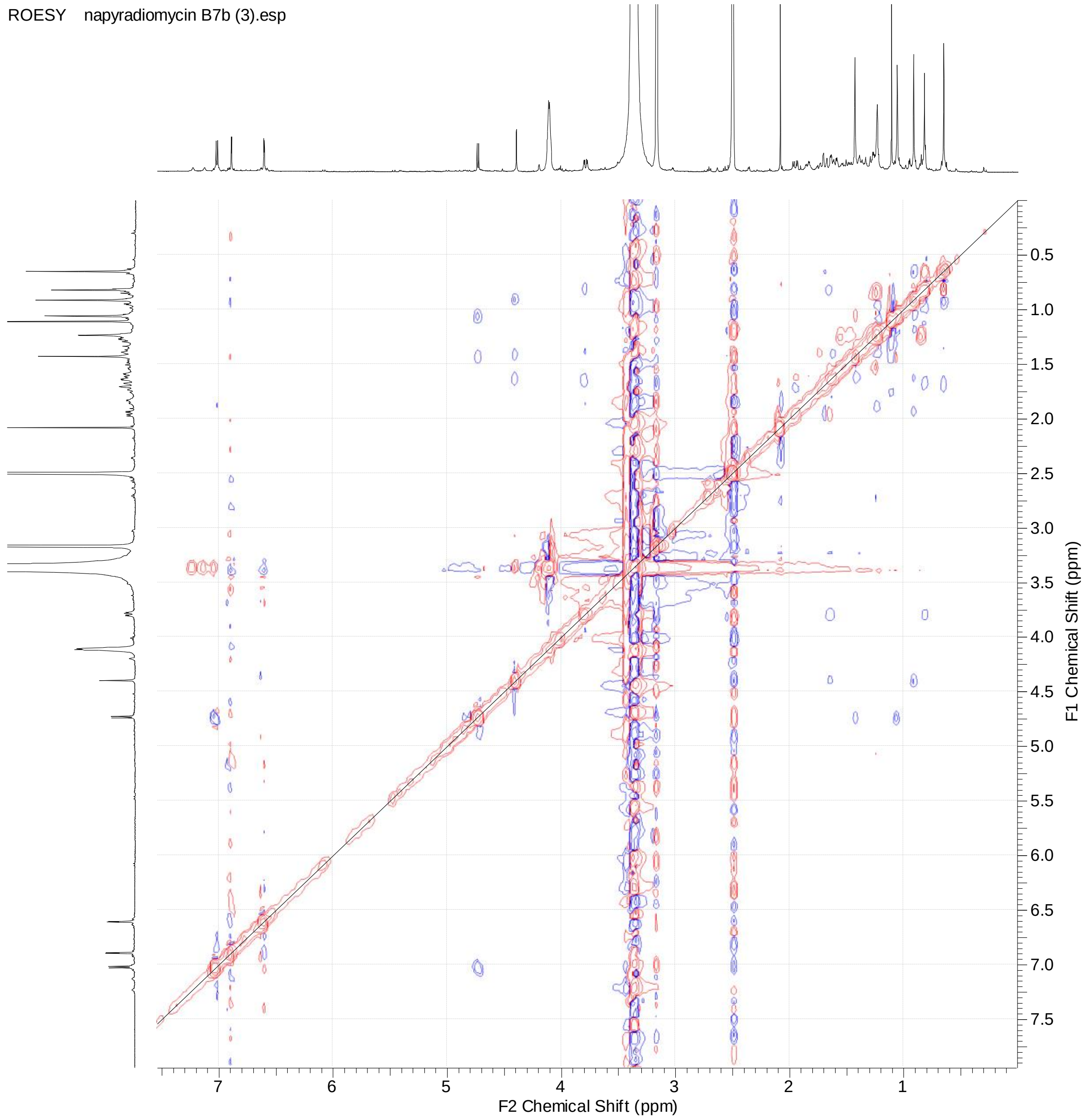

Figure S25. ROESY of compound **3**.

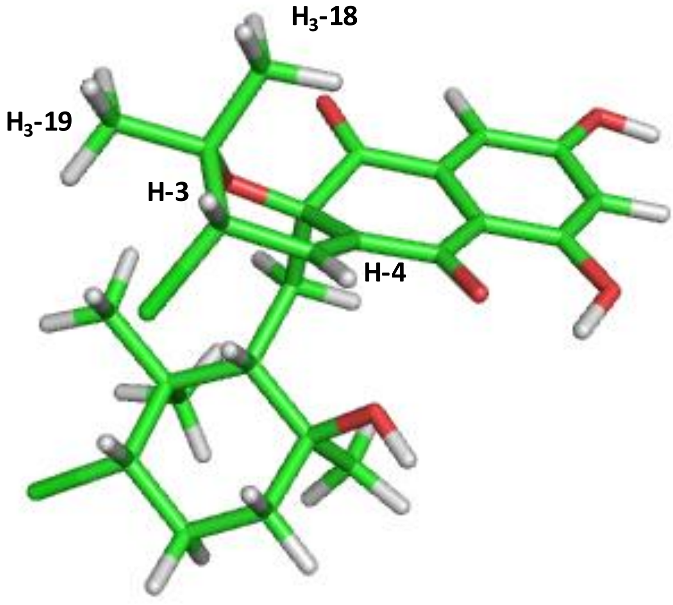

Figure S26. Energy minimized molecular model of compound **3**.

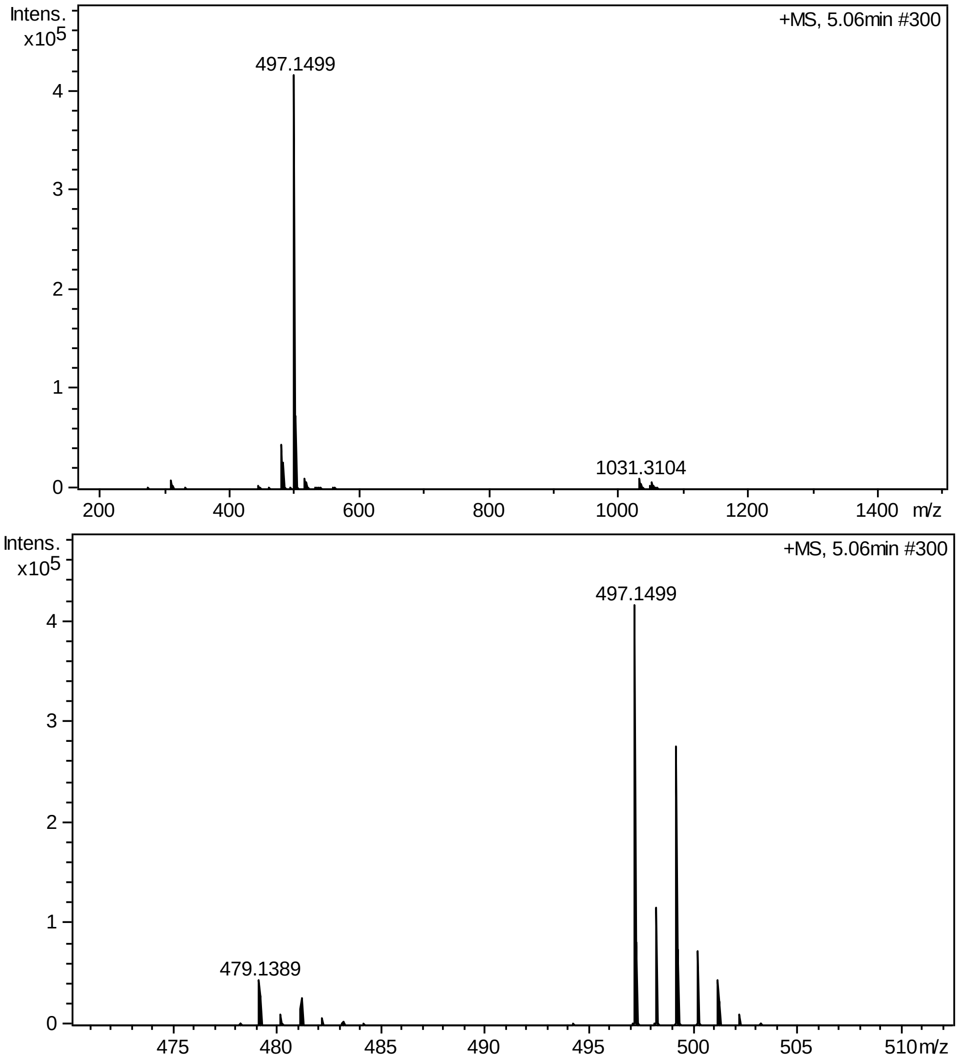

(**A**)

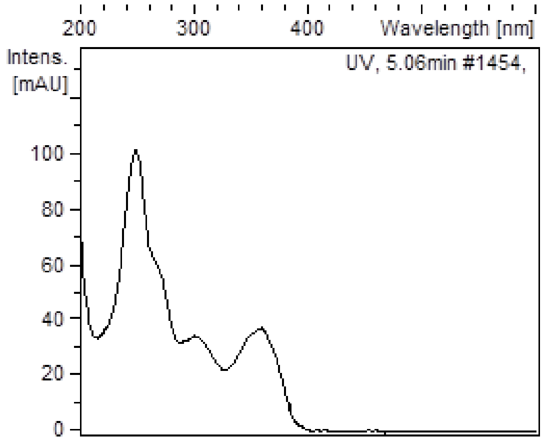

(**B**)

Figure S27. Electrospray-time of flight (ESI-TOF) (**A**) and UV (**B**) spectra of compound **4**.

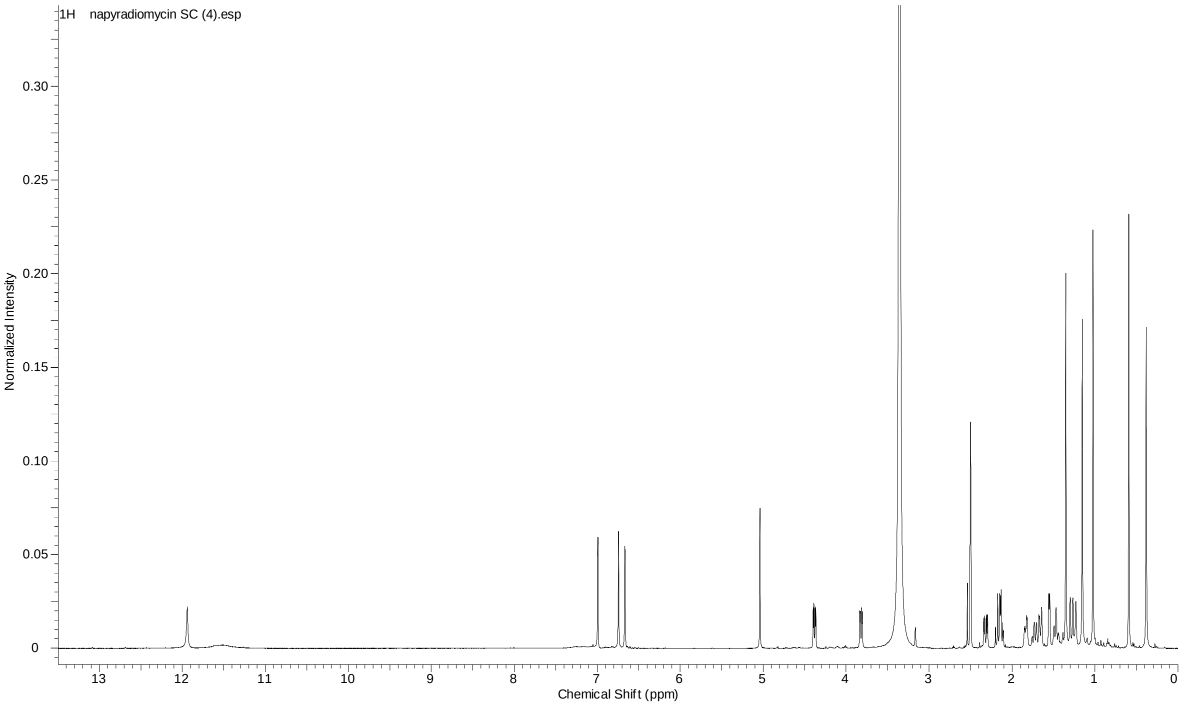

Figure S28. ^{1}H NMR (DMSO_d6, 500 MHz) of compound **4**.

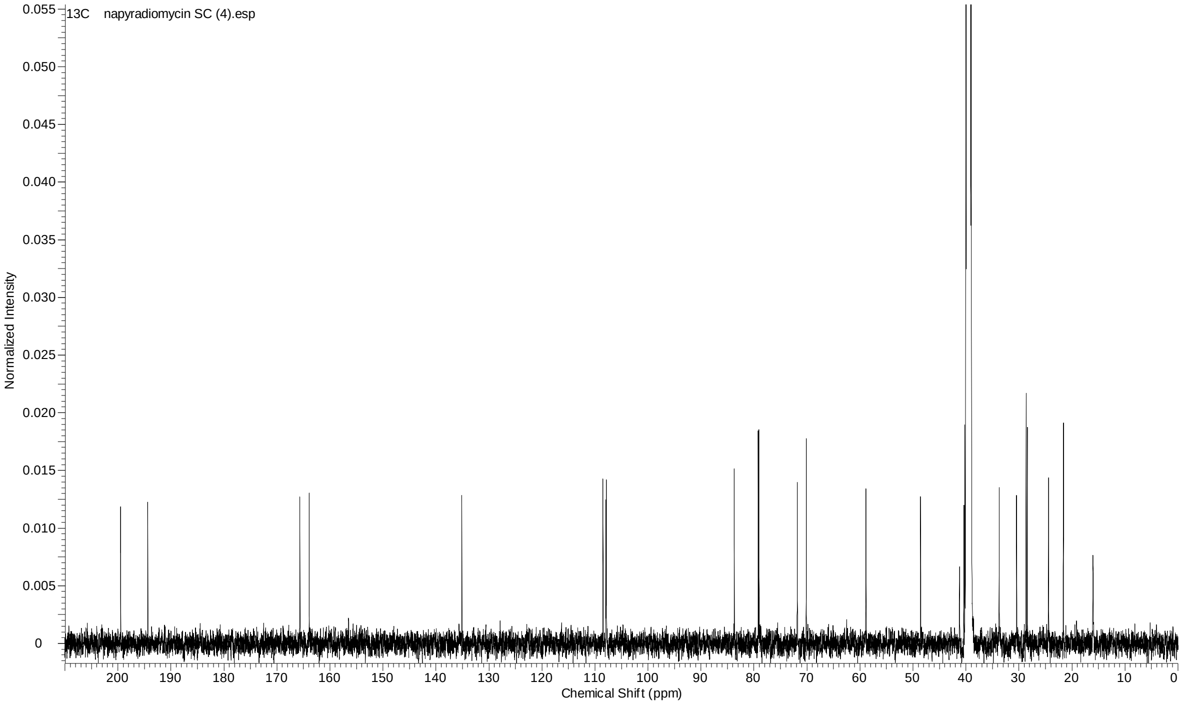

Figure S29. ^{13}C NMR (DMSO_d6, 500 MHz) of compound **4**.

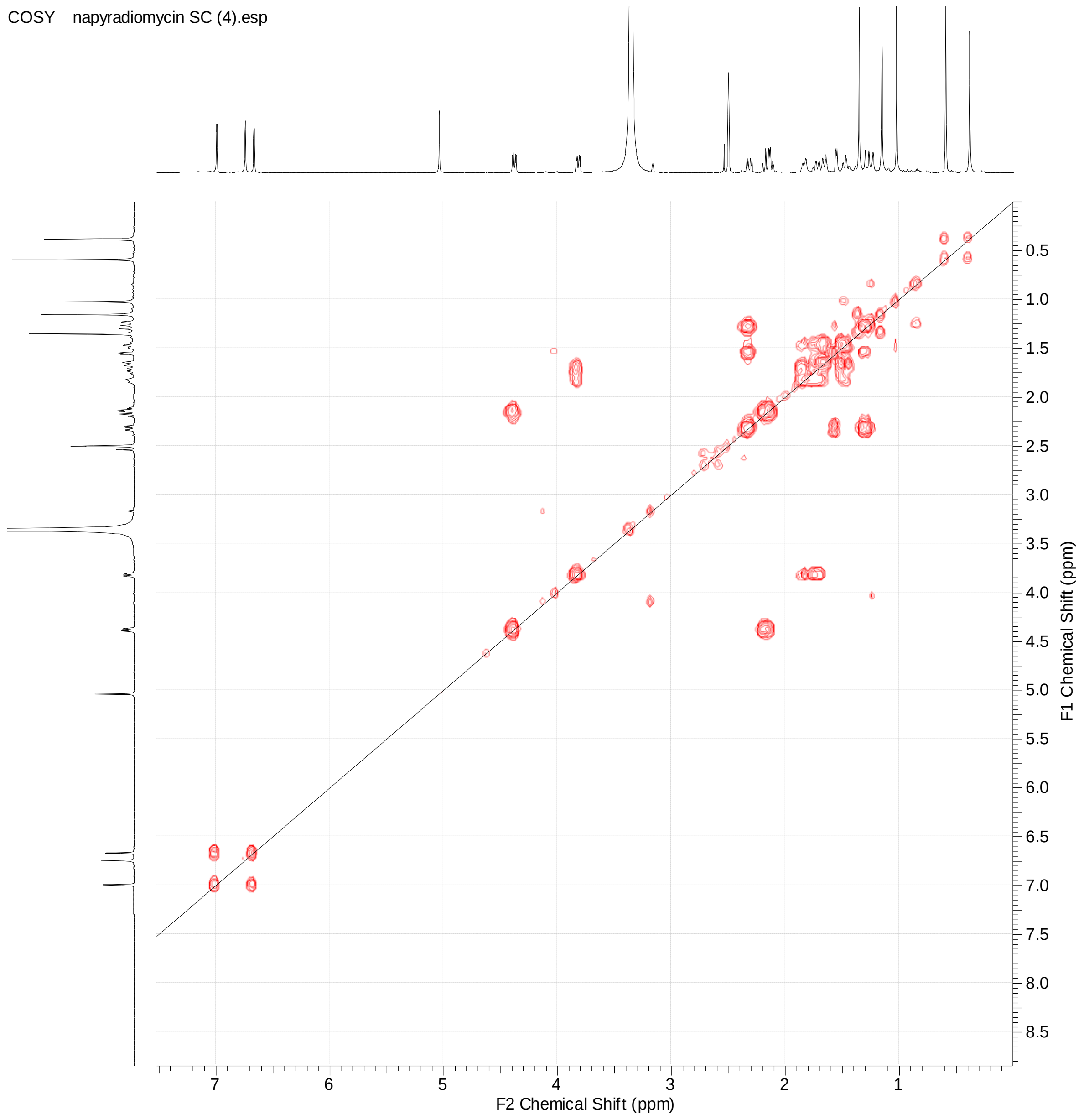

Figure S30. COSY of compound **4**.

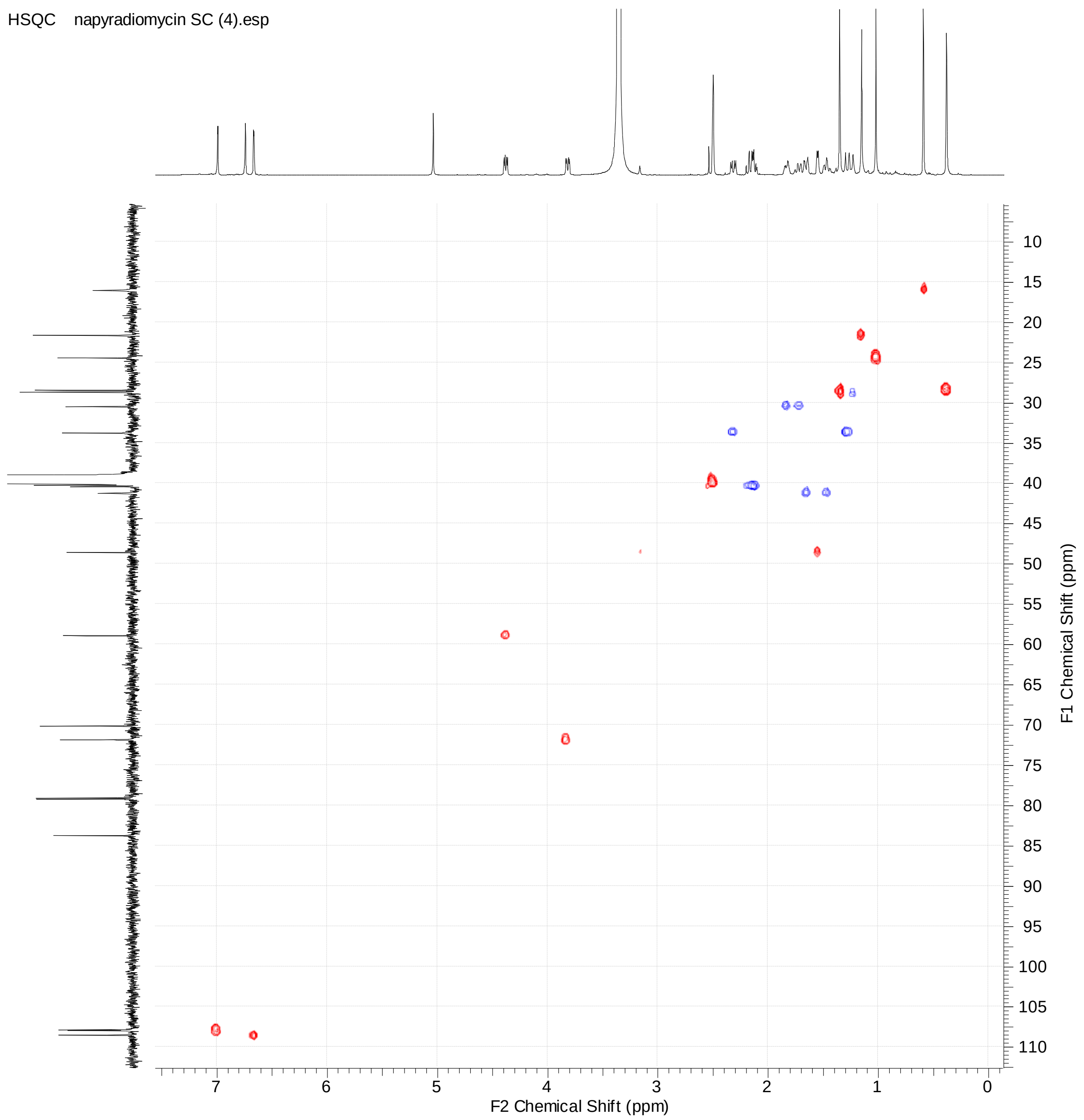

Figure S31. HSQC spectrum of compound **4**.

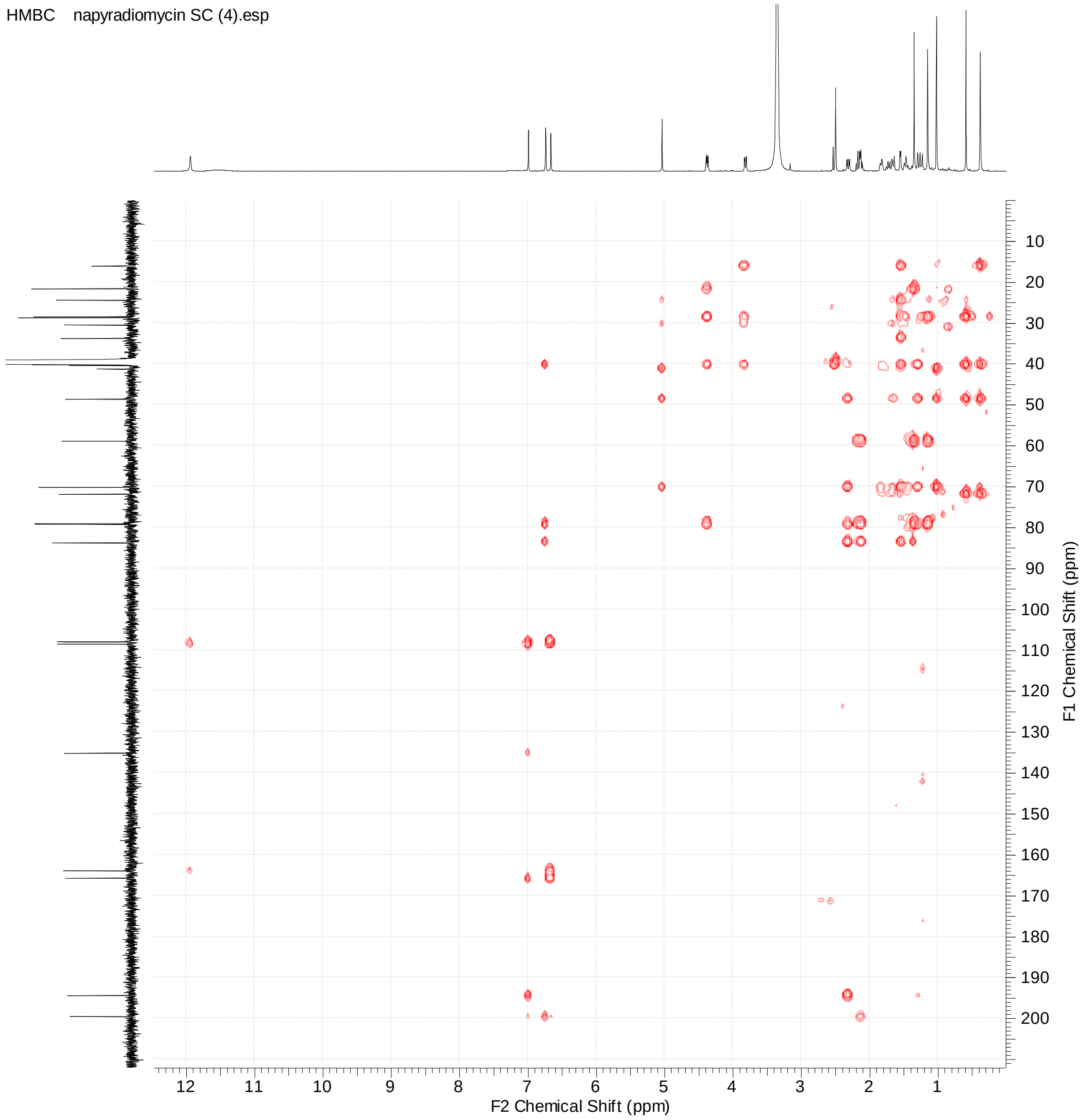

Figure S32. HMBC of compound **4**.

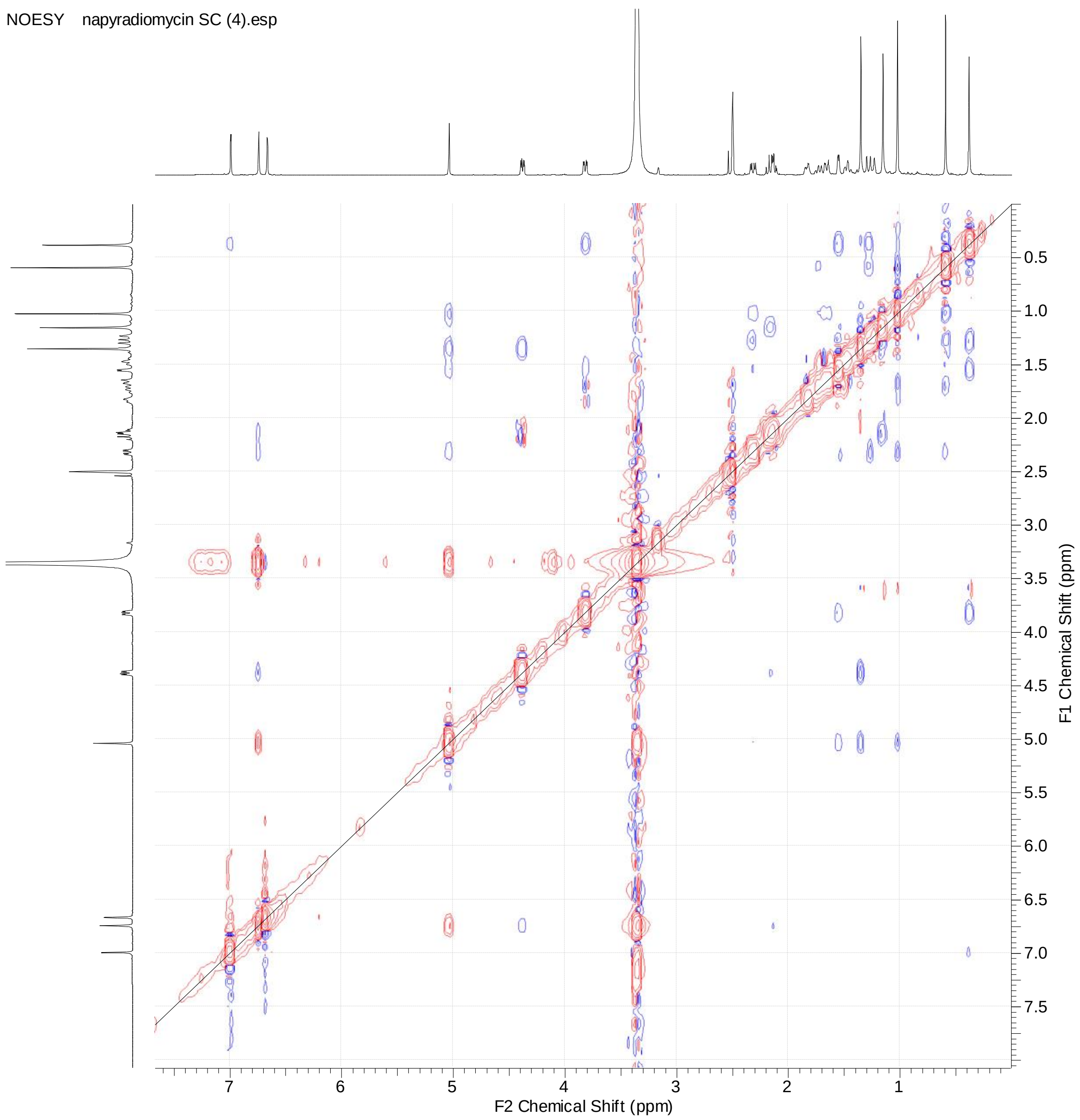

Figure S33. NOESY of compound **4**.

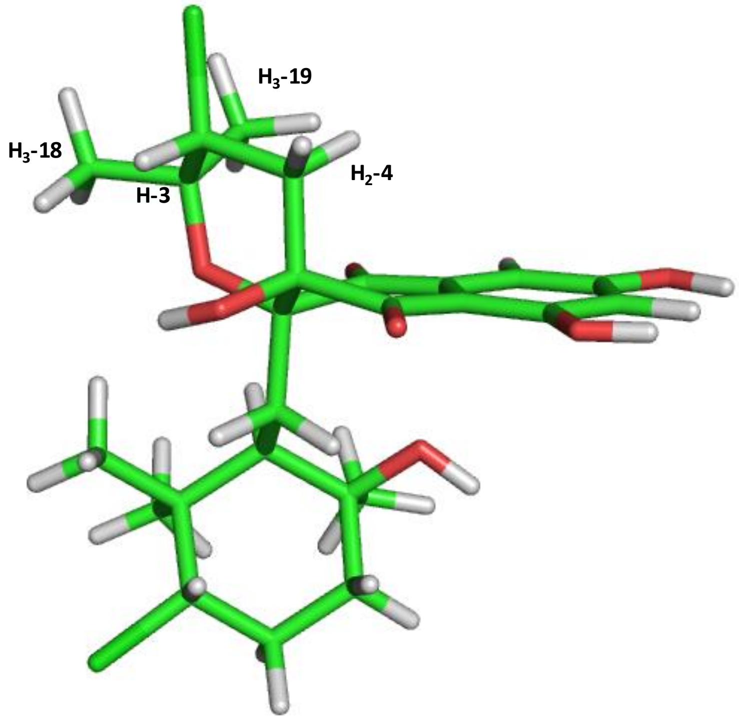

Figure S34. Energy minimized molecular model of compound **4**.

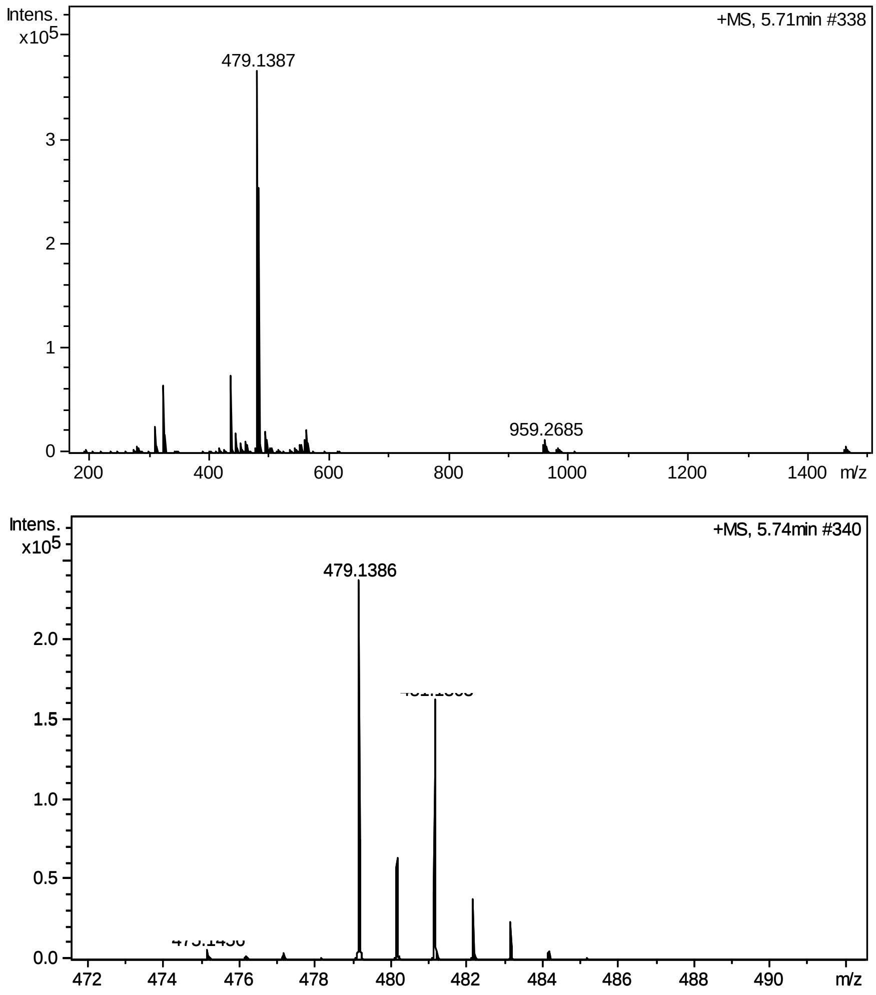

(**A**)

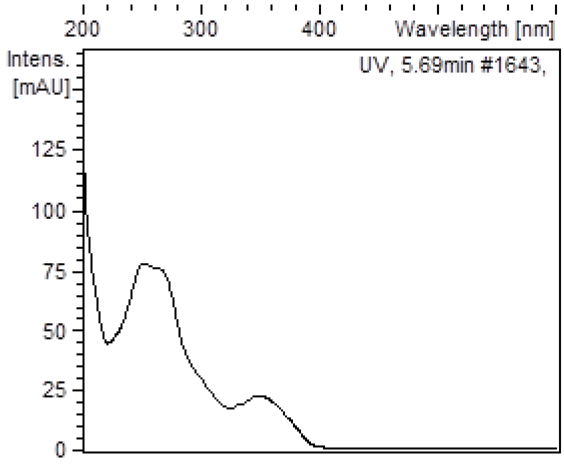

(**B**)

Figure S35. Electrospray-time of flight (ESI-TOF) (**A**) and UV (**B**) spectra of compound **5**.

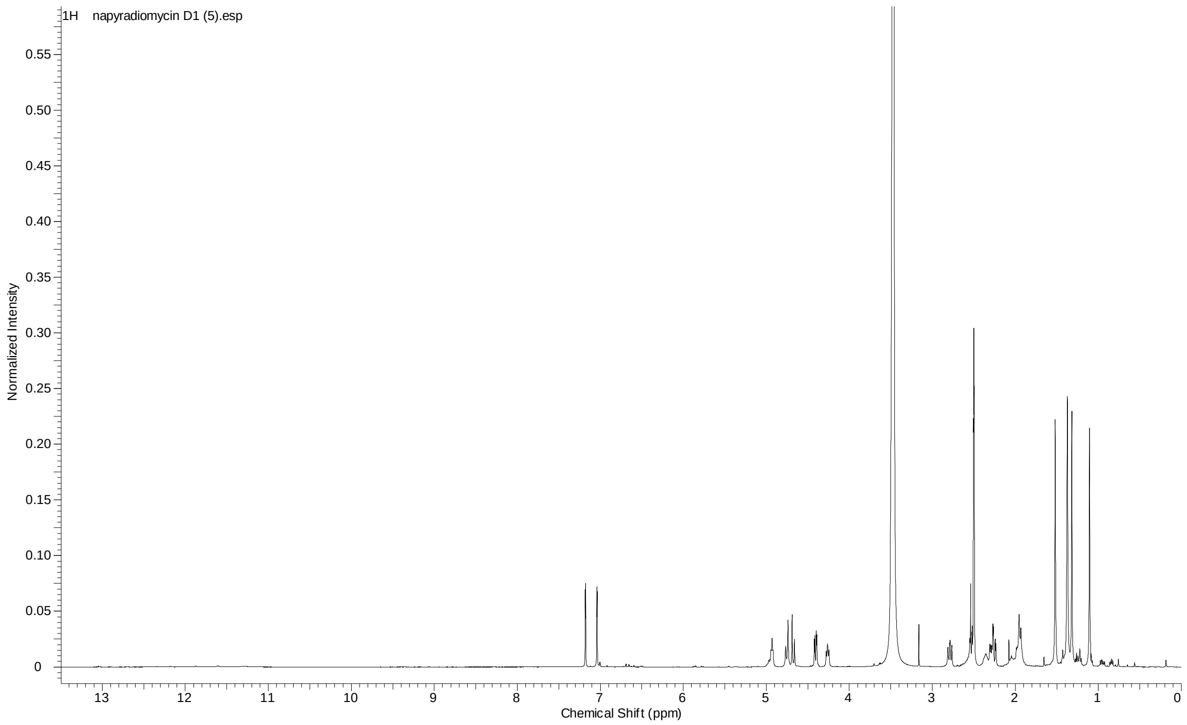

Figure S36. ^{1}H NMR (DMSO_d$_6$, 500 MHz) of compound **5**.

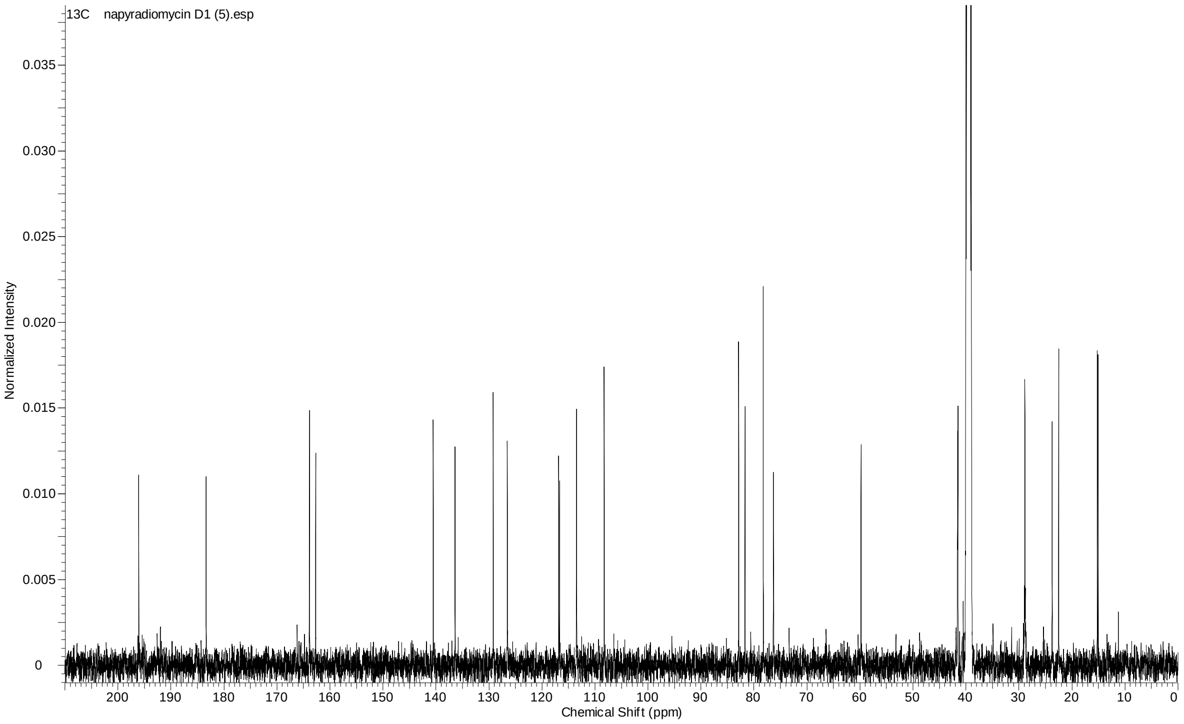

Figure S37. ^{13}C NMR (DMSO_d$_6$, 500 MHz) of compound **5**.

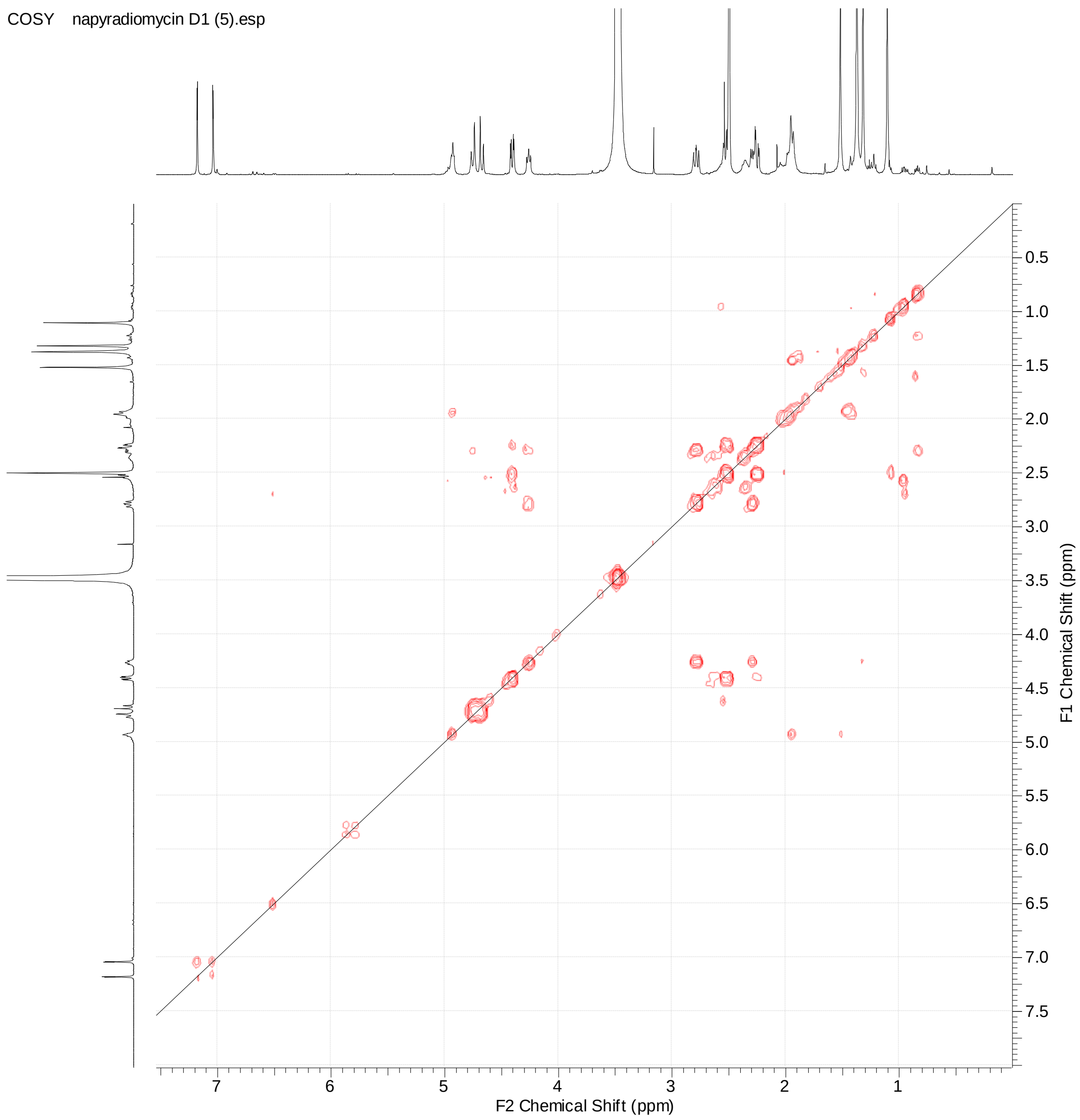

Figure S38. COSY of compound **5**.

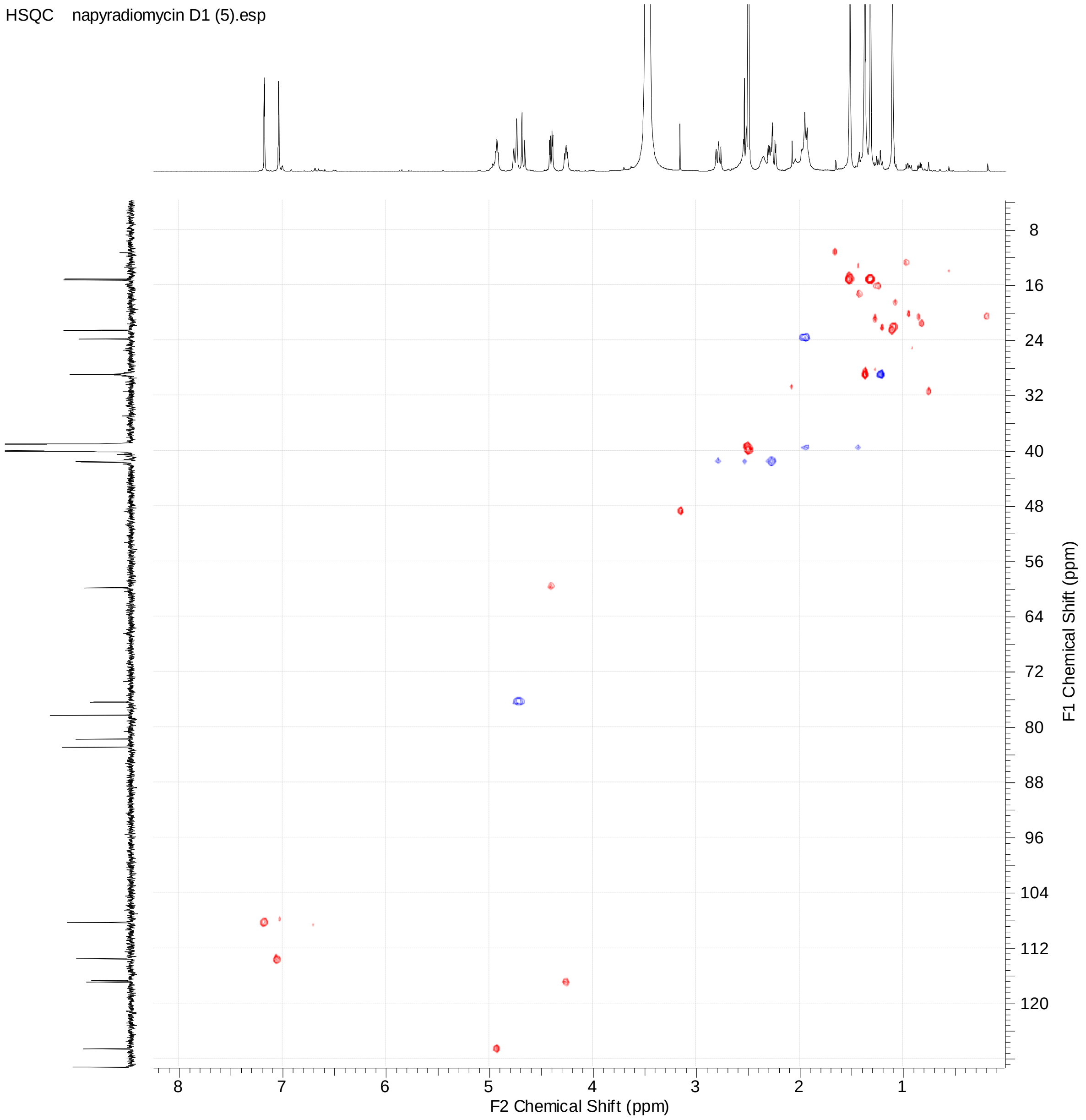

Figure S39. HSQC spectrum of compound **5**.

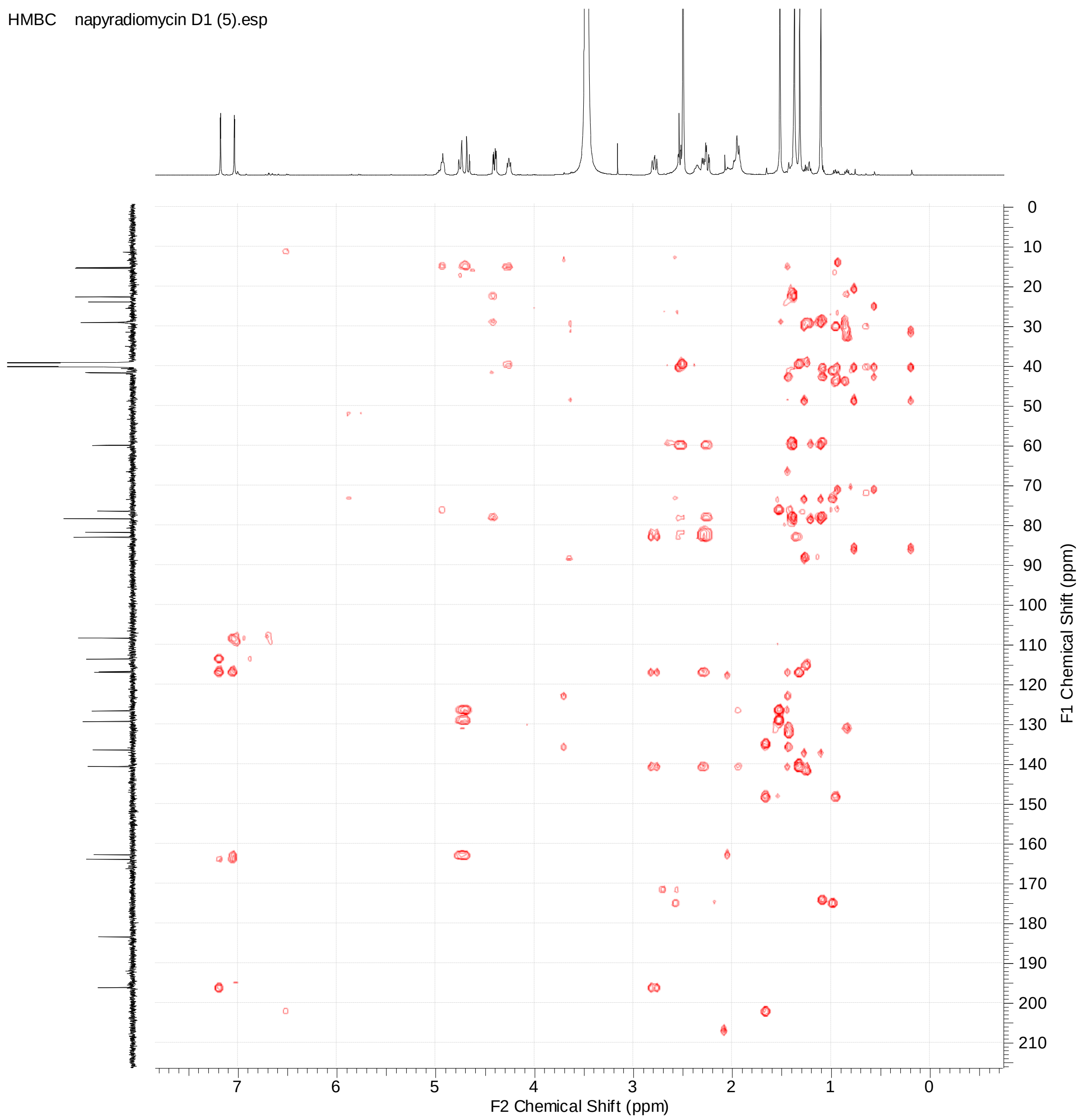

HMBC napyradiomycin D1 (5).esp
F2 Chemical Shift (ppm)
F1 Chemical Shift (ppm)
7
6
5
4
3
2
1
0
10
20
30
40
50
60
70
80
90
100
110
120
130
140
150
160
170
180
190
200
210

(a)

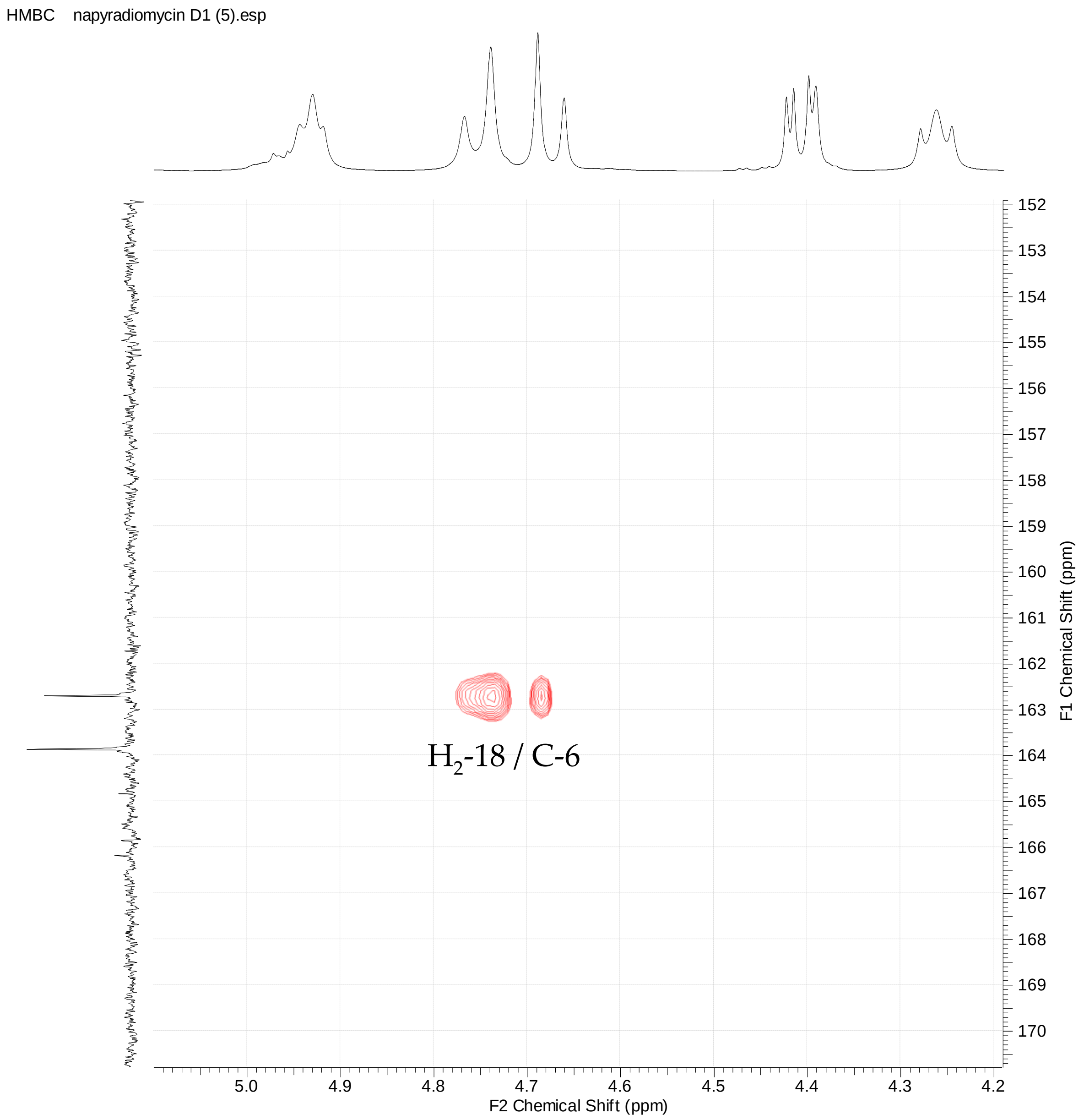

(b)

Figure S40. (a**)** HMBC of compound **5**. (b) Key HMBC correlation supporting the the etherification of the 18-hydroxy group in **5.**

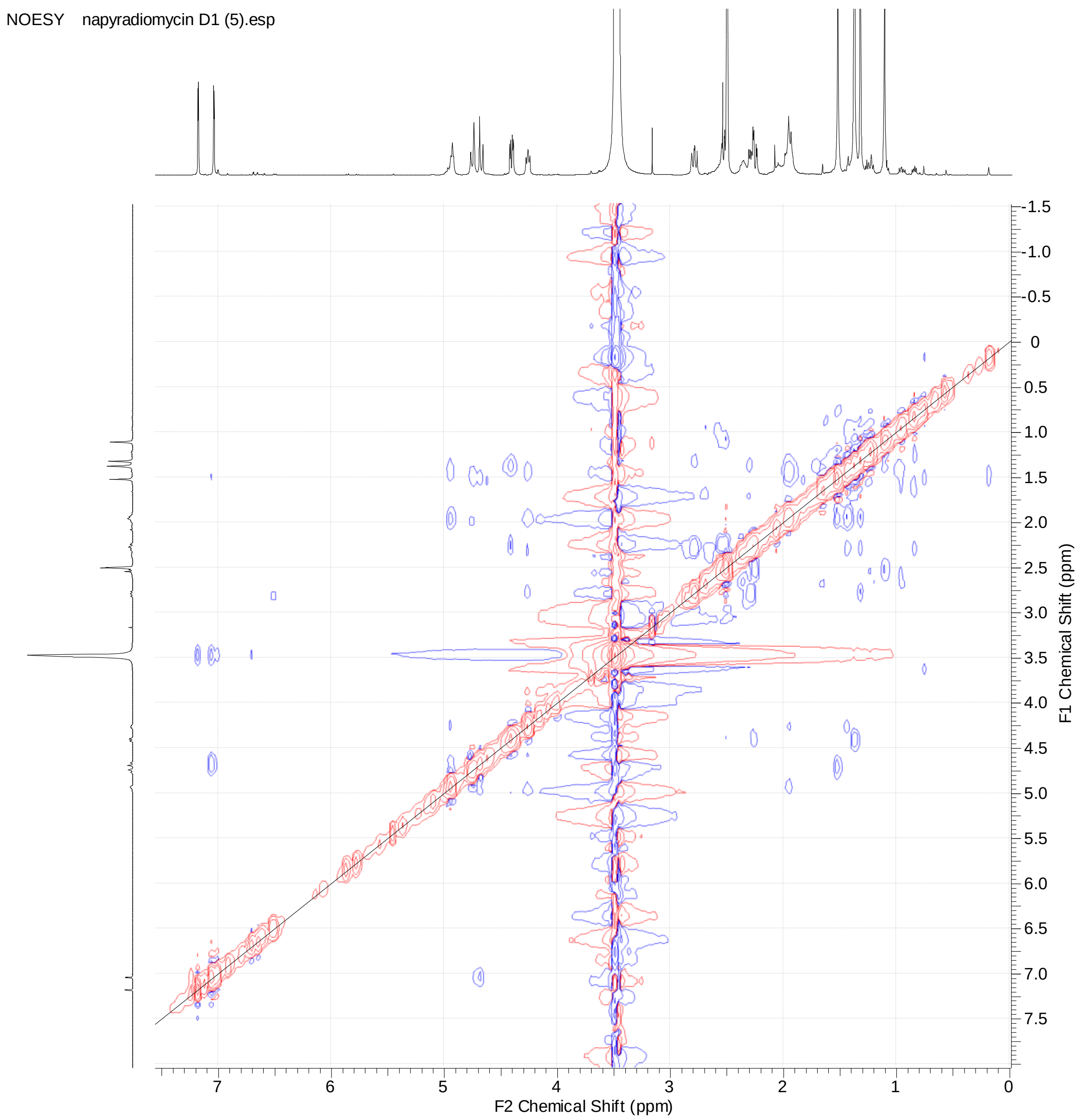

Figure S41. NOESY of compound **5**.

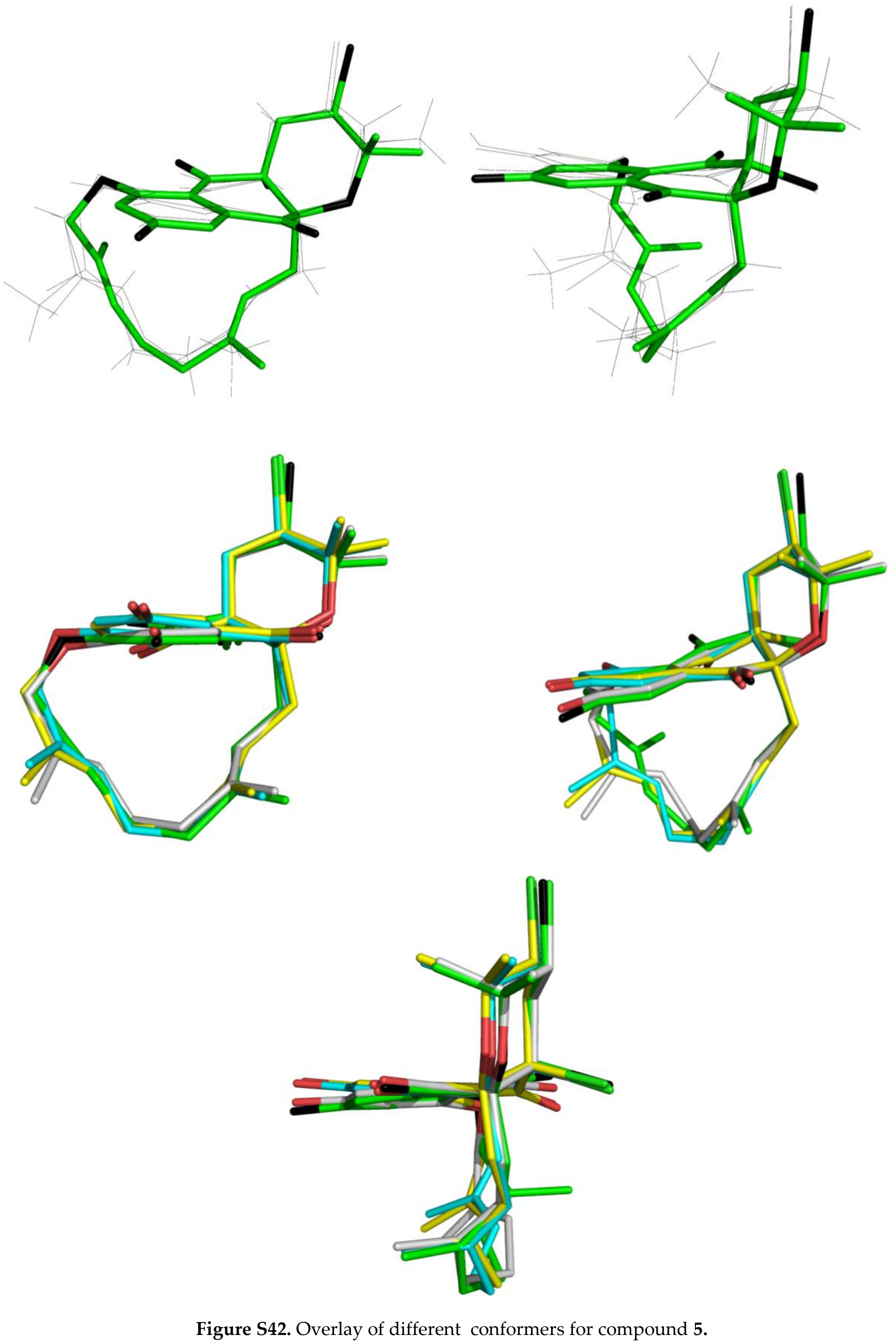

Figure S42. Overlay of different conformers for compound **5.**

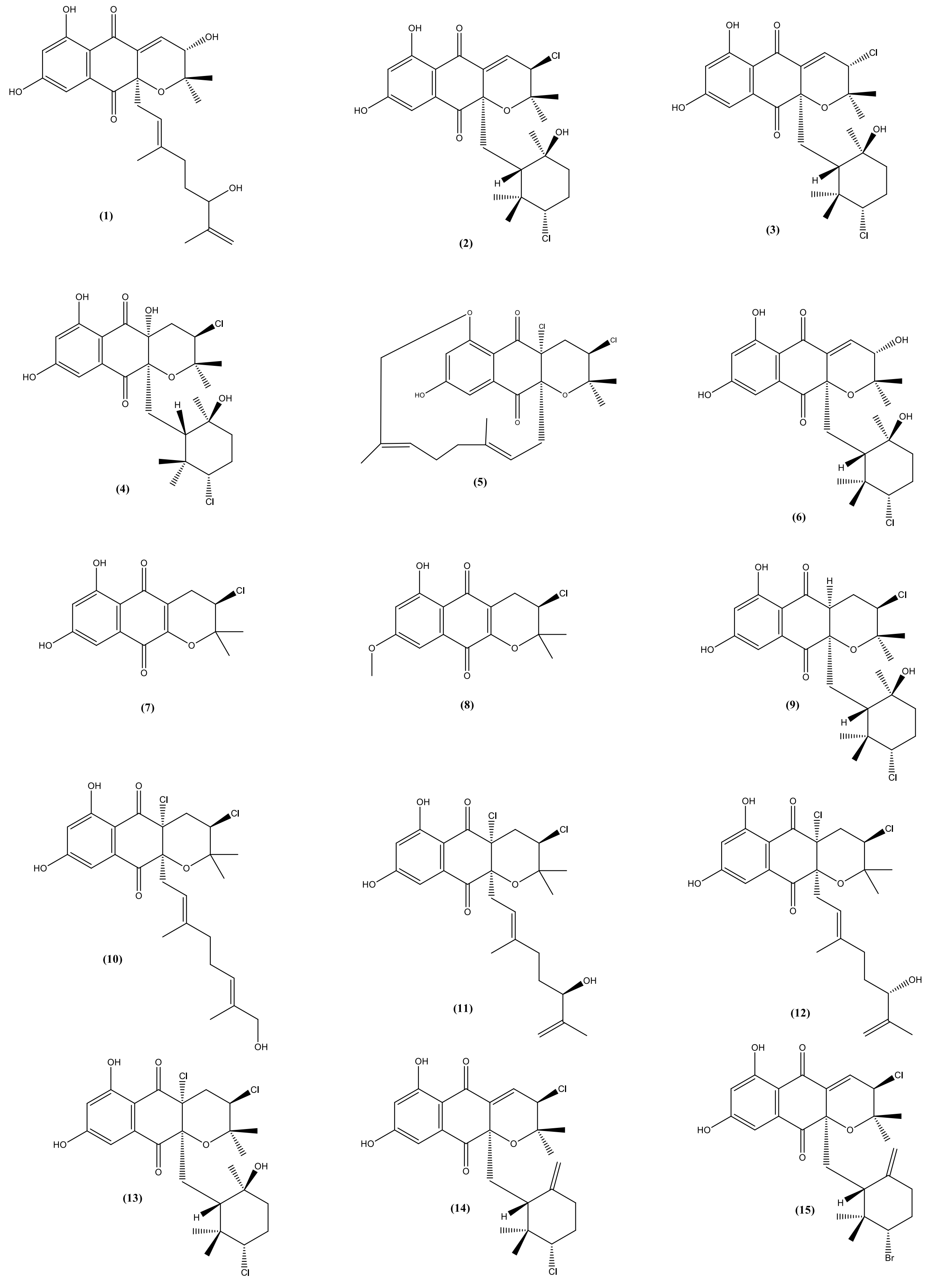

Figure S43. Napyradiomycins metabolites isolated from CA-271078 (**1**-**15**)
